# Ethanol Regulation of Serum Glucocorticoid Kinase 1 Expression in DBA2/J Mouse Prefrontal Cortex

**DOI:** 10.1371/journal.pone.0072979

**Published:** 2013-08-22

**Authors:** Blair N. Costin, Seth M. Dever, Michael F. Miles

**Affiliations:** 1 Virginia Commonwealth University Alcohol Research Center, Virginia Commonwealth University, Richmond, Virginia, United States of America; 2 Department of Pharmacology and Toxicology, Virginia Commonwealth University, Richmond, Virginia, United States of America; 3 Center for Study of Biological Complexity, Virginia Commonwealth University, Richmond, Virginia, United States of America; 4 Department of Neurology, Virginia Commonwealth University, Richmond, Virginia, United States of America; Max Planck Institute of Psychiatry, Germany

## Abstract

**Background:**

We previously identified a group of glucocorticoid-responsive genes, including Serum Glucocorticoid kinase 1 (Sgk1), regulated by acute ethanol in prefrontal cortex of DBA2/J mice. Acute ethanol activates the hypothalamic pituitary adrenal axis (HPA) causing release of glucocorticoids. Chronic ethanol dysregulates the HPA response in both humans and rodents, possibly contributing to important interactions between stress and alcoholism. Because *Sgk1* regulates ion channels and learning and memory, we hypothesized that *Sgk1* contributes to HPA-dependent acute and adaptive neuronal responses to ethanol. These studies characterized acute and chronic ethanol regulation of *Sgk1* mRNA and protein and their relationship with ethanol actions on the HPA axis.

**Results:**

Acute ethanol increased *Sgk1* mRNA expression in a dose and time dependent manner. Three separate results suggested that ethanol regulated Sgk1 via circulating glucocorticoids: acute ethanol increased glucocorticoid receptor binding to the *Sgk1* promoter; adrenalectomy blocked ethanol induction of *Sgk1* mRNA; and chronic ethanol exposure during locomotor sensitization down-regulated HPA axis activation and *Sgk1* induction by acute ethanol. SGK1 protein had complex temporal responses to acute ethanol with rapid and transient increases in Ser422 phosphorylation at 15 min. following ethanol administration. This activating phosphorylation had functional consequences, as suggested by increased phosphorylation of the known SGK1 target, N-myc downstream-regulated gene 1 (NDRG1). After repeated ethanol administration during locomotor sensitization, basal SGK1 protein phosphorylation increased despite blunting of *Sgk1* mRNA induction by ethanol.

**Conclusions:**

These results suggest that HPA axis and glucocorticoid receptor signaling mediate acute ethanol induction of *Sgk1* transcription in mouse prefrontal cortex. However, acute ethanol also causes complex changes in SGK1 protein expression and activity. Chronic ethanol modifies both SGK1 protein and HPA-mediated induction of *Sgk1* mRNA. These adaptive molecular responses of glucocorticoid-responsive gene expression and SGK1 in prefrontal cortex may contribute to mechanisms underlying behavioral responses to chronic ethanol exposure.

## Introduction

Although alcohol dependence is a complex disease that develops over many years and includes cycles of withdrawal, craving, and relapse, acute responses to ethanol have predictive validity in terms of risk for high levels of ethanol intake in animal models and alcoholism in humans [1,2]. Therefore, defining the cellular mechanisms underlying acute responses to ethanol has significant biomedical implications.

Ethanol acutely activates the hypothalamic adrenal pituitary (HPA) axis leading to glucocorticoid release from the adrenal glands [3]. Glucocorticoid hormones are the final step in activation of the HPA axis and are known to function in the biological response to stress and circadian activity [4,5]. Glucocorticoids are also well known to regulate gene expression [6]. In alcohol dependence, the HPA axis is dysregulated in both humans [7,8] and rodents [9–11], but the consequences of this dysregulation remain unclear.

Our laboratory and others have used genome-wide expression profiling to identify gene networks functioning in acute and chronic behavioral responses to ethanol [12–17]. We previously identified a group of genes prominently regulated by acute ethanol in the prefrontal cortex (PFC) of DBA2/J (D2) mice [12]. Contained in this group were well-characterized glucocorticoid responsive genes including *FK506 binding* protein *5* (*Fkbp5*) and Serum Glucocorticoid Kinase 1 (Sgk1) [18,19]. *Sgk1* is a glucocorticoid responsive gene that regulates ion channel function, cell survival, and is involved in synaptic plasticity, learning and memory [20–24].

*Sgk1* has multiple transcript and protein isoforms generated though alternative promoter utilization, splicing, translation and post-translational modifications [25,26]. It is known that there are 5 isoforms of *Sgk1—4* resulting from translational processing of *Sgk1* and one, *Sgk1.1*, resulting from alternative promoter utilization and splicing [25,26]. Because *Sgk1* is regulated by both glucocorticoids and acute ethanol and is known to regulate ion channel function and synaptic plasticity, we hypothesized that Sgk1 signaling may be an important mechanism underlying acute cellular responses to ethanol in brain and might also play a role in behavioral adaptations with chronic ethanol exposure. We have therefore performed a detailed analysis on ethanol regulation of Sgk1 from the transcriptional to protein level. Our results indicate a complex regulation of *Sgk1* transcription, protein abundance and post-translational modification following acute and chronic ethanol treatment.

## Material and Methods

### Ethics Statement

All procedures were approved by Virginia Commonwealth University Institutional Animal Care and Use Committee under protocol number AM10332 and followed the NIH Guide for the Care and Use of Laboratory Animals (NIH Publications No. 80–23, 1996).

### Animals

Mice were maintained in a temperature-controlled room (23°C±1) with 12 h light/dark cycles and free access to standard chow (Harlan Teklad #7912, Madison, WI, United States) and water. Cages and bedding (Harlan Sani-chips, #7090A, Harlan, Teklad, Madison, WI, United States) were changed weekly. All tests were carried out between 0900 and 1200 h. All mice were DBA2/J mice from Jackson Laboratories (Bar Harbor, ME, United States) purchased at 10-13 weeks of age and group housed 4/cage. Adrenalectomized (ADX) and sham mice were treated at Jackson and delivered following recovery from surgery. ADX mice were supplemented with 1% w/v saline solution in their drinking water. All mice were allowed to habituate to the animal facility for at least 1 week prior to testing.

### Adrenalectomy

All adrenalectomies and sham procedures were conducted using aseptic and atraumatic surgical techniques at Jackson Laboratories and were approved by the institution’s Animal Care and Use Committee. According to documentation provided by Jackson Laboratories, surgeries were performed using the lateral abdominal approach. Left and right adrenal glands were removed through separate incisions. The animals were anesthetized using tribromoethanol, carprofen was administered for analgesia and the surgical site was prepared. The animals were placed in right lateral recumbency and a 5-8mm incision was made parallel and ventral to the spine and midway between the last rib and iliac crest. The underlying muscle was opened and the adrenal gland was located cranial to the left kidney. The adrenal gland was grasped with ring forceps and exteriorized. The adrenal and adjacent adipose tissues were excised. The incisions in the abdominal wall and skin were closed separately. Bupivacaine was applied topically to the incision site for local analgesia. Skin closure material was removed prior to shipment. The same procedure was followed for excision of the right adrenal gland except the skin incision was made immediately caudal to the last rib. This adjustment was necessary to accommodate for the more cranial location of the right adrenal gland relative to the left in the abdomen. Surgeries were performed throughout the day from 7: 30 am to 2:30 pm.

### Drugs

All drugs were administered intraperitoneally (i.p.). Saline solutions were 0.9% w/v sterile saline. Ethanol solutions were prepared from 200-proof absolute anhydrous ethanol (Pharmco-Aaper brand, Brookfield, CT, United States). Ethanol was administered at 20% v/v in 0.9% saline.

### Experimental Testing

Mice were habituated to injections with saline in their home cage for 2 days prior to experimental testing. If behavioral testing was performed, mice were allowed a 1-hour acclimation period to the behavioral room prior to testing. All locomotor activity was measured immediately following injection with either saline or ethanol, during a 10-minute session in locomotor activity chambers (Med-Associates, model ENV-515; St. Albans, VT, United States).

#### Exp. 1 – Time course and dose response analysis of Sgk1 and Sgk1.1 expression following acute ethanol administration

For the time course experiment, six groups of mice (n = 4 each) were administered either saline or 4 g/kg ethanol and groups of animals, one saline treated group and one ethanol treated group, were harvested 2, 4 and 8 hours following drug administration. For the dose response experiment, three groups of mice (n = 8) were administered saline, 2 g/kg ethanol or 4 g/kg ethanol and harvested 4 hours following injection and brain micropunch dissections performed.

#### *Exp*. *2 – Sgk1 levels following ethanol sensitization*

Animals (n = 4) received one of three treatments: saline-saline (SS), saline-ethanol (SE) or ethanol-ethanol (EE) (Table **1**). On conditioning days 3-13, animals received daily injections in their home cages of either saline (SS, SE) or 2.5 g/kg ethanol (EE). On test day 14 the SS group received saline and the SE and the EE groups received 2.0 g/kg ethanol and were placed in locomotor activity chambers for testing x 10 minutes. They were then returned to their home cages and four hours following drug administration, brains were harvested and brain micropunch dissections performed.

**Table 1:** Outline of experimental design for ethanol sensitization.

**Group**	**Days 1-2**	**Days 3-13**	**Day 14**
	**Habituation**	**Conditioning**	**Activity Test**
SS	Saline	Saline	Saline
SE	Saline	Saline	Ethanol 2.0 g/kg
EE	Saline	Ethanol 2.5 g/kg	Ethanol 2.0 g/kg
ES	Saline	Ethanol 2.5 g/kg	Saline

#### Exp. 3 – Corticosterone levels following ethanol sensitization

Animals (n=6) received one of four treatments: saline-saline (SS), ethanol-saline (ES), saline-ethanol (SE) or ethanol-ethanol (EE) (Table **1**) with doses, schedules and behavioral testing as with Exp. 2. One hour following the last drug administration, animals were harvested and trunk blood collection performed.

#### Exp 4 – Effects of ADX on Sgk1 induction following ethanol administration

ADX and Sham animals (n=11-15) received either 4 g/kg ethanol or saline. Mice were harvested four hours following ethanol or saline administration and brain micropunch dissections performed for RNA isolation and Q-rtPCR. To ensure that mice were in fact adrenalectomized, a separate group of ADX and SHAM mice (n = 4-5) were administered either saline or acute ethanol. One hour following the last drug administration, animals were harvested and trunk blood collection performed for determination of corticosterone levels.

#### Exp. 5 – Glucocorticoid receptor binding to Sgk1 promoter following acute ethanol administration

Mice (n = 8/group, 48 total) were treated with saline or 4 g/kg ethanol and harvested via cervical dislocation 1 hour following drug administration. Following cervical dislocation, the brain was removed, the olfactory bulbs were separated from the brain and a cut was made just rostral to the optic chiasm to collect the frontal pole of the brain. Tissue was then processed for DNA or chromatin isolation for chromatin immunoprecipiation (ChIP) assays.

#### Exp. 6 – SGK1 phosphoSerine (S422) and SGK1 Levels following acute ethanol administration

Mice (n = 12, 6 animals treated with saline, 6 treated with 4 g/kg ethanol) were administered saline or ethanol and harvested 15 minutes, 1 hour, 2 hours, 4 hours, 8 hours and 24 hours following drug administration and brain micropunch dissections performed for protein isolation and Western blotting.

#### Exp. 7 – SGK1 phosphoSerine (S422) Levels following chronic ethanol administration (ethanol sensitization)

Animals (n=6-8/group) received one of four treatments: saline-saline (SS), ethanol-saline (ES), saline-ethanol (SE) or ethanol-ethanol (EE) (Table **1**) with doses, schedules and behavioral testing as with Exp. 3. Fifteen minutes following drug administration, animals were harvested and brain micropunch dissections performed for protein isolation and Western blotting.

#### Exp. 8 – SGK1 and NDRG1 Basal Expression in Neurons and Oligodendrocytes

Mice (n = 2) were deeply anesthetized with sodium pentobarbital (180 mg/kg i.p.) and perfused transcardially with normal saline followed by 10 ml of 4% paraformaldehye. Brains were removed and post-fixed overnight in 4% paraformaldehyde, cryoprotected in 30% sucrose, and frozen using dry ice cooled 2-methylbutane. Tissue was stored at -80 °C until immunohistochemistry was performed.

### Brain Micropunch Dissection

At designated times following ethanol or saline administration in experiments 1, 2, 4, 6 and 7 brain tissue was collected. Collection of brain tissue occurred exactly as described in Kerns et al. [12]. The medial PFC dissection contained tissue from dorsal and ventral anterior cingulate and some secondary motor cortex.

### RNA Isolation and Quantitative Real Time Polymerase Chain Reaction (Q-rtPCR)

RNA was isolated from PFC tissue samples using the RNeasy Protect Mini Kit (Qiagen, Valencia, CA, United States) according to the manufacturer’s instructions. RNA concentration and quality was assessed by Experion automated electrophoresis (BioRad, Hercules, CA, United States). cDNA was generated from 1 µg total RNA by reverse transcription with the iScript CDNA kit (Bio-Rad, Hercules, CA, United States) following the manufacturer’s instructions. Q-rtPCR was performed using the iCycler iQTM system (Bio-Rad, Hercules, CA, United States) according to the manufacturer’s instructions for SYBR Green I-based detection. Quantification of gene expression levels was determined based on the threshold cycle for each well using the provided software and all results were normalized to multiple reference genes using Genorm as described in Vandesompele et al. [27]. Primers used were as follows, *Sgk1* (Forward- CGTCAAAGCCGAGGCTGCTCGAAGC and Reverse- GGTTTGGCGTGAGGGTTGGAGGAC), *Sgk1.1* (Forward- ATGCCAACATCCTGACCAA and Reverse- TGCTGGCAATCTTCTGAATAAA), *Glyceraldehyde 3-phosphate dehydrogenase* (*Gapdh*) (Forward-TTCCAGTATGACTCCACTCACGG and Reverse-TGAAGACACCAGTAGACTCCACGAC), *protein phosphatase 2, regulatory* subunit *B, alpha* (*Ppp2r2a*) (Forward-ATCTCTCACCCTTGCCCTTT and Reverse-CCCATTTTGTGTGCTTTCGT), *ubiquitin-like domain containing CTD phosphatase* (*Ublcp1*) (Forward-ATGACAGGGACAGGACAAGC and Reverse-TACAATGACACCCGACTGGA), *NDUFV1 NADH dehydrogenase* (*ubiquinone*) *flavoprotein 1* (*Ndufv1*) (Forward- GACCGTGCTAATGGACTTCG and Reverse-GGCATCTCCCTTCACAAATC), *nuclear receptor* subfamily *3,* group *C,* member *1* (glucocorticoid receptor) (*Nr3c1*) (Forward-AAGAGACAAACGAGAGTCCTTGG and Reverse- GTGTCCGGTAAAATAAGAGGCTT, and *Fkbp5* (Forward-GCCGACTGTGTGTGTAATGC and Reverse-

CACAATACGCACTTGGGAGA).

### Blood collection and Radioimmunoassay (RIA)

One hour following ethanol or saline administration in Exp. 3 and Exp. 4, trunk blood was collected from individual mice. Serum was isolated by centrifugation at 2500 x g for 15 minutes and stored at -80°C until RIA assay. A RIA containing I^125^ labeled corticosterone (MP Biomedicals, Cleveland, OH, United States) was performed according to the manufacturer’s instructions.

### Chromatin Immunoprecipitation (ChIP) Assay

Frontal poles from 8 D2 mice in Exp. 5 were combined to make one individual sample and the ChIP analyses were performed using the magnetic bead-based Chip-IT Express Enzymatic kit following the manufacturer’s instructions for fresh tissue (Active Motif, Carlsbad, CA, United States). Brieﬂy, the tissue was cross-linked with 1% formaldehyde for 10 min, sheared enzymatically for 2 hours and the chromatin immunoprecipitated with the indicated antibodies: rabbit anti-Glucocorticoid Receptor (sc-1004, Santa Cruz Biotechnology, Santa Cruz, CA, United States) and rabbit anti-immunoglobulin G (IgG) (2729, Cell Signaling, Danvers, MA, United States). Following immunoprecipitation, the chromatin was eluted from the magnetic beads, the cross-links were reversed, and the protein was digested. Samples were then subjected to a DNA clean-up step prior to Q-rtRCR using the QIAquick PCR Purification Kit (Qiagen, Valencia, CA, United States) according to the manufacter’s instructions. The resulting DNA fragments in the range of 150 to 500 bp were analyzed by Q-rtRCR using a pair of primers (Forward- ACCCCTGCTCCCTCTAACTC and Reverse-GCGGAAATAAGTCTCTGCTCT) spanning the glucocorticoid response element (GRE) in the *Sgk1* promoter region. For Q-rtRCR, SsoAdvanced™ SYBR® Green Supermix (BioRad, Hercules, CA, United States) was used according to the manufacturer’s instructions.

### Western Blotting

While frozen, PFC tissue from animals in Experiments 6 and 7 was homogenized using a Dounce Tissue Homogenizer (Fisher Scientific, Waltham, MA, United States) and then suspended in Lithium Dodecyl Sulfate Loading Buffer (Invitrogen, Grand Island, NY, United States) containing Halt protease and phosphatase inhibitors (Thermo, Fisher Scientific, Waltham, MA, United States) and sonicated. Western blotting was performed using the XCell Surelock Mini-Cell kit (Invitrogen, Grand Island, NY, United States) according to the manufacturer’s instructions. SGK1 blots were probed with rabbit anti-SGK1 (ab59337, AbCam, Cambridge, MA, United States) and rabbit anti-GAPDH (ab9485, AbCam, Cambridge, MA, United States). phospho-SGK1 (pSGK1) blots were probed with rabbit anti-SGK1 phospho S422 (ab55281, AbCam, Cambridge, MA, United States) (Experiments 6 and 7) and goat anti-SGK1 phospho T256 (sc-16744, Santa Cruz Biotechnology, Santa Cruz, CA, United States) (Exp. 6), then stripped using Restore Western Blot Stripping Buffer (Thermo Scientific, Rockford, IL, United States) and re-probed with rabbit anti-SGK1 (ab59337, AbCam, Cambridge, MA, United States). phospho-N-myc downstream-regulated 1 gene 1 (pNDRG1) blots were probed with rabbit anti-NDRG1 phospho S330 (ab124713, AbCam, Cambridge, MA, United States), then stripped and re-probed with rabbit anti-NDRG1 (ab63989, AbCam, Cambridge, MA, United States). Blots were also probed with the secondary antibodies IRDye goat anti-rabbit 680, IRDye goat anti-rabbit 800, and IRDye donkey anti-goat 800 antibodies (Li-cor Biosciences, Lincoln, NE, United States). Western blot imaging was performed and images were quantified using infrared imaging (Odyssey infrared imager, Li-cor Biosciences, Lincoln, NE, United States) [28].

### Immunohistochemistry

Tissue from Exp. 8 was cut into 30 micron-thick coronal sections using a cryostat. Sections were permeabilized with 0.2% Triton X-100, and immunolabeled. Primary antibodies to SGK1 (ab59337, AbCam, Cambridge, MA, United States), NR3C1 (sc-1004, Santa Cruz Biotechnology, Santa Cruz, CA, United States), NDRG1 (ab63989, AbCam, Cambridge, MA, United States), MAP2 (ab5392, AbCam, Cambridge, MA, United States), and CNPase (ab50739, AbCam, Cambridge, MA, United States) were used. SGK1, NR3C1 and NDRG1 were visualized with Alexa Fluor 594 (A11012, Invitrogen, Grand Island, NY, United States), and MAP2 and CNPASE with Alexa Fluor 488 (A-11039, Invitrogen, Grand Island, NY, United States) conjugated secondary antibodies. Lastly sections were stained with DAPI to reveal their nuclei. The PFC region of sections was imaged using a Zeiss LSM 700 confocal laser scanning microscope. Images were extracted using ZEN 2011 (blue edition) imaging software (Carl Zeiss Microscopy, LLC, Thornwood, NY, USA).

### Statistics

Data were expressed as mean ± SEM and analyzed parametrically. Data were analyzed with analysis of variance (ANOVA) using appropriate between- and within subject factors. All post hoc comparisons were made using Student Newman-Keul’s test. Values of *p* < 0.05 were considered statistically significant.

## Results

### Time course and dose response analysis of Sgk1 and Sgk1.1 expression following acute ethanol administration

**Figure 1:**
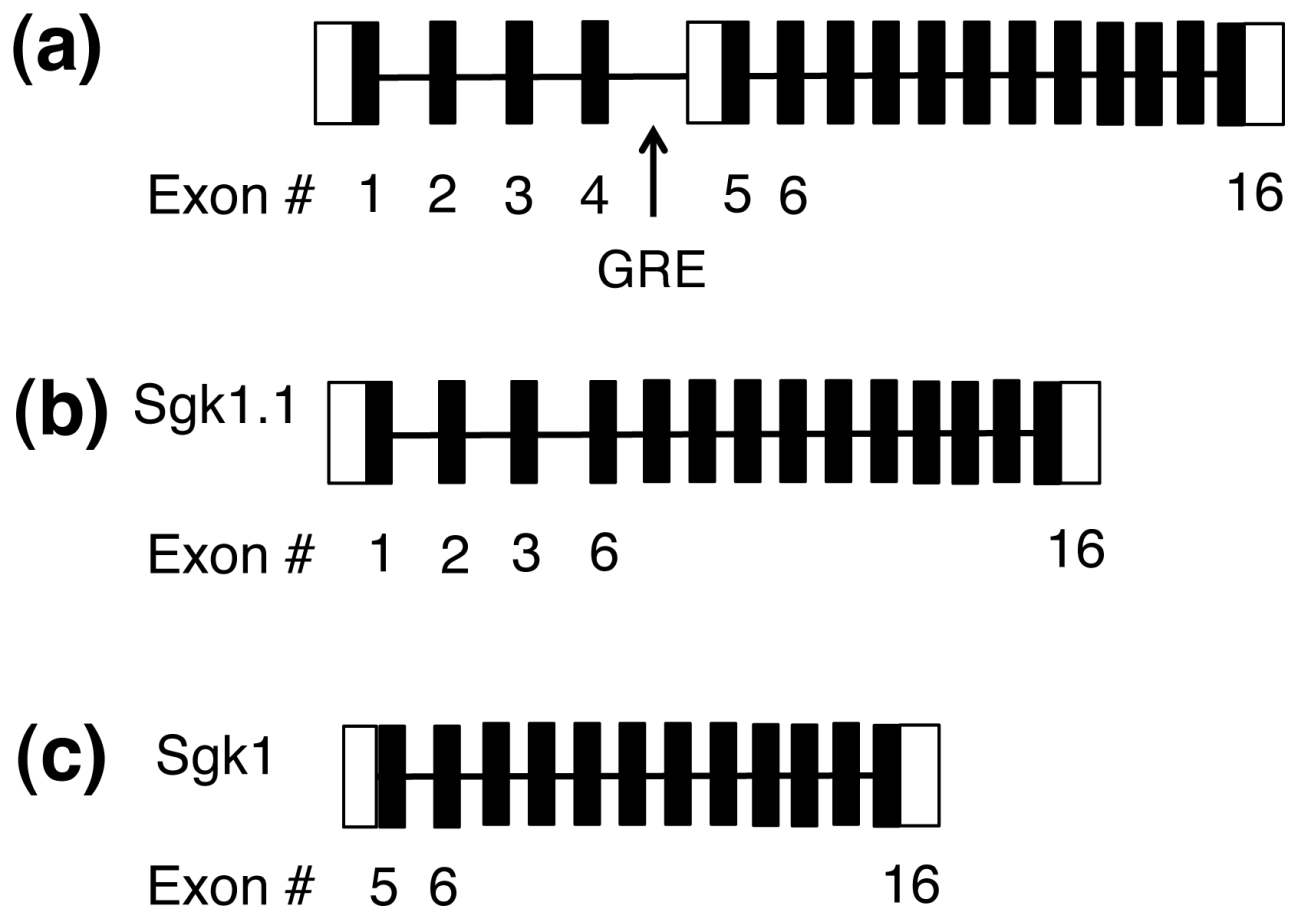
Schematic of the SGK1 gene and N termini of Sgk1.1 versus Sgk1. (a) White boxes indicate 5’ and 3’ untranslated regions of Sgk1.1 and Sgk1, respectively; black boxes represent exons and the line represents introns. The SGK1 promoter contains a GRE. (b) Exons of the N termini of Sgk1.1. (c) Exons of the N termini of Sgk1.

**Figure 2:**
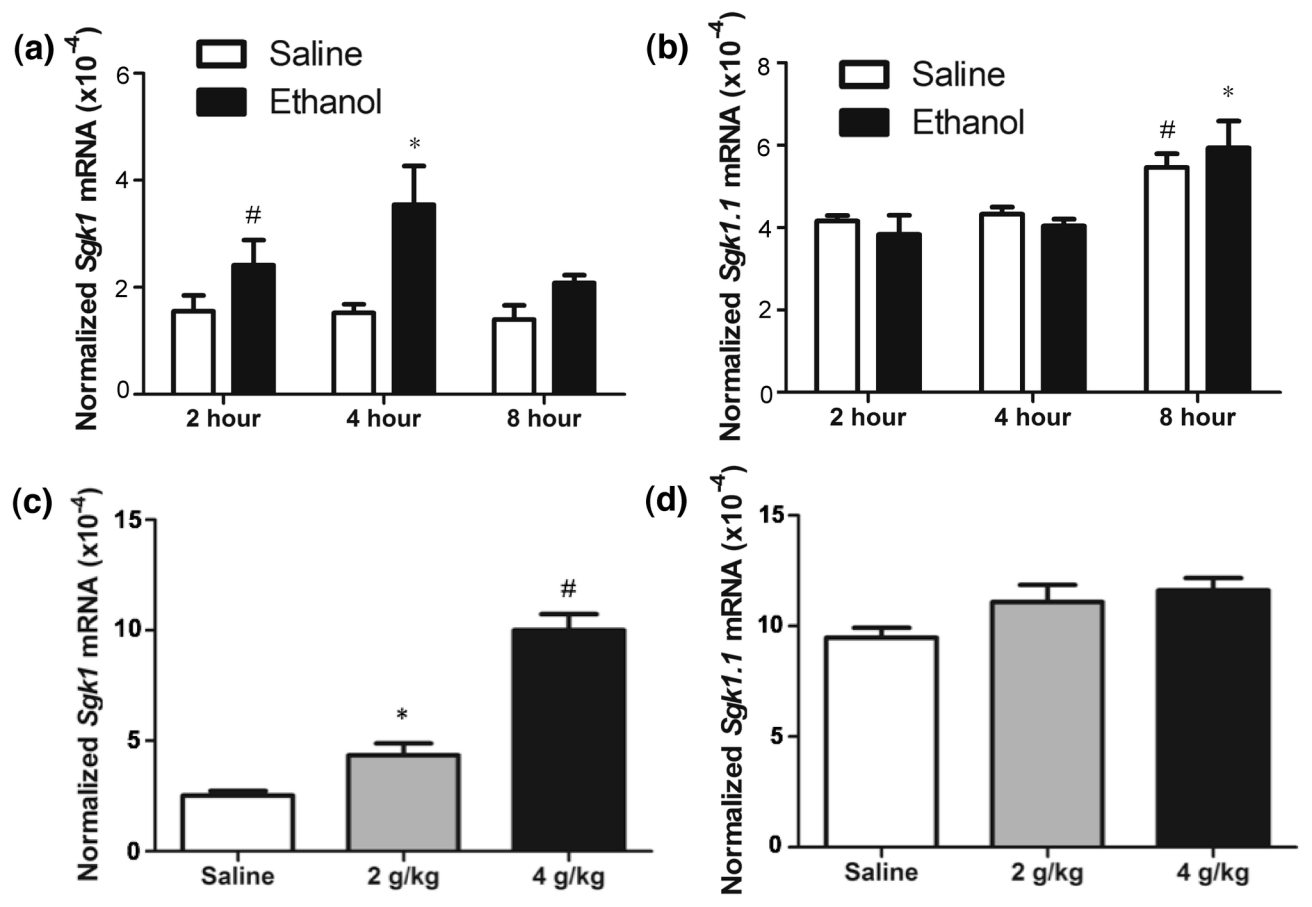
Acute ethanol time course and dose response analysis for *Sgk1* and *Sgk1.1* expression. Q-rtPCR analysis of *Sgk1* and *Sgk1.1*. Panels show: (a) *Sgk1* following 4 g/kg ethanol administration. * p < 0.05 versus all saline treated animals and 8 hour ethanol group, # p < 0.05 versus 8 hour saline group; (b) *Sgk1.1* levels following 4 g/kg ethanol administration. * p < 0.05 versus ethanol and saline animals at 2 or 4 hours following ethanol administration, # p < 0.05 versus 2 hour ethanol group; (c) Ethanol dose response for *Sgk1* 4 hours following ethanol administration. * p < 0.05 versus saline treated animals, # p < 0.05 versus ethanol and saline treated animals (d) *Sgk1.1* ethanol dose response at 4 hours.

Because our prior microarray studies and the studies of other labs would have used probes that recognize cDNA regions common to all *Sgk1* isoforms (Figure **1a–c**), we performed Q-rtPCR studies to determine which *Sgk1* isoform was specifically regulated by ethanol, *Sgk1.1* (Figure **1b**) or *Sgk1* (Figure **1c**). Q-rtPCR was used to evaluate *Sgk1* and *Sgk1.1* levels 2, 4 and 8 hours following ethanol or saline administration. Prior to performing these studies on ethanol, we evaluated the effects of injection stress on Sgk1 expression as a crtical control. We compared *Sgk1* levels in the PFC of D2 mice basally (0 hour time point) to D2 mice harvested 2, 4 and 8 hours following saline injections. Saline injections did not significantly alter Sgk1 levels at any time point compared to basal Sgk1 levels (Figure **S1**). Because we saw no significant effects of saline injections on Sgk1 expression, we did not include a 0 hour time point in the remainder of our studies. In evaluating *Sgk1* levels, a two-way ANOVA showed an overall effect of treatment, ethanol versus saline (*F*_1,17_ = 16.44, *p* < 0.01), but no overall effect of time and no significant treatment x time interaction (Figure **2a**). A one-way ANOVA showed a significant effect of treatment (*F*_5,22_ = 4.51, *p* < 0.01) and post-hoc analysis indicated that 4 hours following ethanol treatment *Sgk1* levels were significantly increased compared to all saline treated animals and animals treated with ethanol 8 hours prior to harvest (Figure **2a**). In addition, *Sgk1* levels were significantly increased in animals harvested 2 hours following 4 g/kg ethanol administration compared to those harvested 8 hours following saline administration (Figure **2a**). This indicates that *Sgk1* levels were significantly increased 2 and 4 hours following 4 g/kg ethanol administration, returning to basal levels by 8 hours.

A two-way ANOVA evaluating *Sgk1.1* levels showed no overall effect of treatment or time and no significant treatment x time interaction (Figure **2b**). However, there were significant increases in Sgk1.1 mRNA at 8 hours following treatment in both the ethanol and saline injected animals (one-way ANOVA, *F*_5,18_ = 5.31, *p* < 0.01), suggesting a general response to the injections themselves. These results suggest that *Sgk1*, not *Sgk1.1*, is the ethanol responsive isoform of *Sgk1*.

To further evaluate which isoform of *Sgk1* was ethanol responsive, Q-rtPCR was used to evaluate *Sgk1* and *Sgk1.1* levels following an ethanol dose response assessment in which animals were administered saline, 2 g/kg and 4 g/kg ethanol. In evaluating *Sgk1* levels, a one-way ANOVA showed a significant effect of treatment (*F*_2,18_= 50.58, *p* < 0.01). Post-hoc analysis indicated significant differences between 4 g/kg ethanol versus saline, 2 g/kg ethanol versus saline and 4 g/kg ethanol versus 2 g/kg ethanol indicating that *Sgk1* is dose dependently increased following ethanol administration (Figure **2c**). Q-rtPCR analysis of *Sgk1.1* transcript levels following saline, 2 g/kg and 4 g/kg ethanol administration showed no significant effects between the 3 groups by one-way ANOVA (Figure **2d**). Once again this indicated that *Sgk1*, not *Sgk1.1*, is the ethanol responsive isoform.

### Sgk1 levels following chronic ethanol sensitization

**Figure 3:**
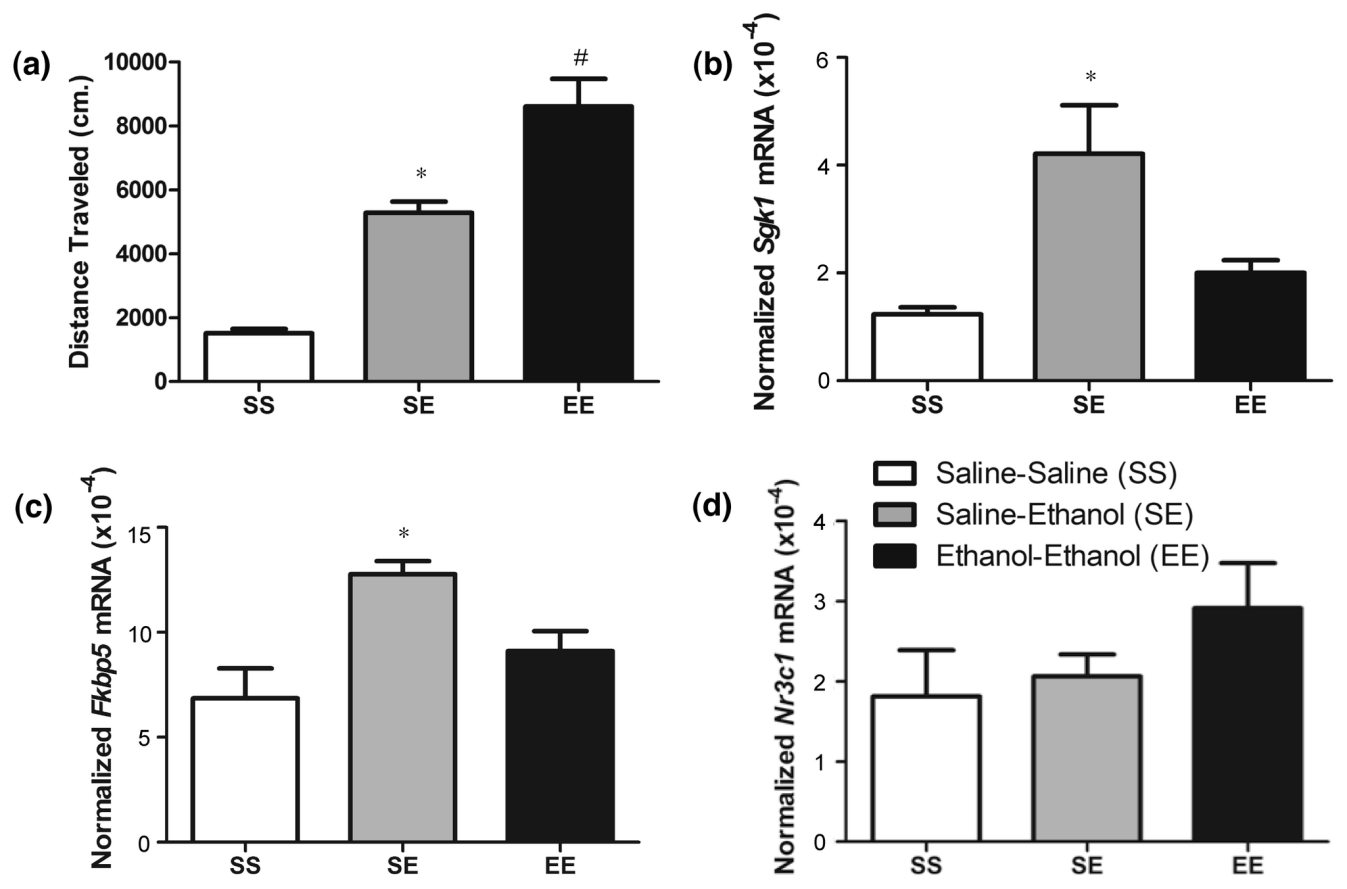
*Sgk1* mRNA expression following ethanol sensitization. Behavioral sensitization followed by Q-rtPCR analysis of *Sgk1* and *Nr3c1*. Panels show: (a) Total locomotor activity (cm/10min.) for saline only (SS), acute ethanol (SE) and ethanol sensitized (EE) groups. * p < 0.05 versus chronic saline (SS), # p < 0.05 versus acute ethanol (SE) (b) *Sgk1* levels in SS, SE and EE treated mice 4 hours following following saline (SS) or ethanol (SE, EE) treatment on day 14. * p < 0.05 versus SS and EE treated animals (c) *Nr3c1* levels in SS, SE and EE treated mice as in panel b.

Prior microarray studies in our lab and others had identified *Sgk1* to be an acute ethanol responsive gene and we confirmed this finding via Q-rtPCR (Figure **2a–d**). However, since we predicted that the HPA axis is involved in ethanol regulation of Sgk1 and chronic ethanol exposure is known to dysregulate the HPA axis, we performed studies to determine if *Sgk1* was regulated following chronic ethanol administration as in locomotor sensitization. To answer this question, we evaluated *Sgk1* and *Fkbp5* (another acute ethanol and glucocorticoid-responsive gene) levels acutely and chronically following ethanol sensitization studies. One-way ANOVA showed an overall effect of treatment on locomotor activity (*F*_2,21_= 41.96, *p* < 0.01) (Figure **3a**). Post-hoc analysis revealed that ethanol-ethanol (EE) treated animals had significantly greater locomotor activity compared to saline-ethanol (SE) and saline-saline (SS) treated animals. In addition, SE treated animals showed greater locomotor activity than SS treated animals.

Q-rtPCR analysis of *Sgk1* mRNA levels (4 hours post injection) in these animals showed an overall significant effect of treatment (one-way ANOVA, *F*_2,9_= 8.76, *p* < 0.01) (Figure **3b**). Post-hoc analysis showed that *Sgk1* levels were significantly increased in animals treated acutely with ethanol compared to SS and EE treated animals. Interestingly, *Sgk1* levels did not differ between SS and EE treated animals. A one-way ANOVA also identified significant differences in *Fkbp5* levels (Figure **3c**). There was an overall significant effect of treatment (*F*_2,9_= 8.07, *p* = 0.01). Post-hoc analysis showed that *Fkbp5* levels were significantly increased in animals treated acutely with ethanol compared to SS and EE treated animals. Like *Sgk1, Fkbp5* levels did not differ between SS and EE treated animals. As additional controls, we also performed PCR analysis of mRNA levels for the glucocorticoid receptor (*NR3c1*) and the non-glucocorticoid responsive *Sgk1.1* isoform of *Sgk1*. A one-way ANOVA identified no significant differences in *Nr3c1* (Figure **3d**) or *Sgk1.1* (Figure **S2**) levels in SS, SE or EE treated animals. These results indicated that *Sgk1* and *Fkbp5*, another ethanol responsive, glucocorticoid responsive gene, are regulated by ethanol acutely, but not following sensitization. Furthermore, *Sgk1*, not *Sgk1.1* is again the ethanol responsive isoform of *Sgk1*. The diminished *Sgk1* response following ethanol sensitization does not appear to be due to down-regulation of glucocorticoid receptor levels, at least as assessed by *Nr3c1* mRNA abundance*.*

### Corticosterone levels following ethanol sensitization

**Figure 4:**
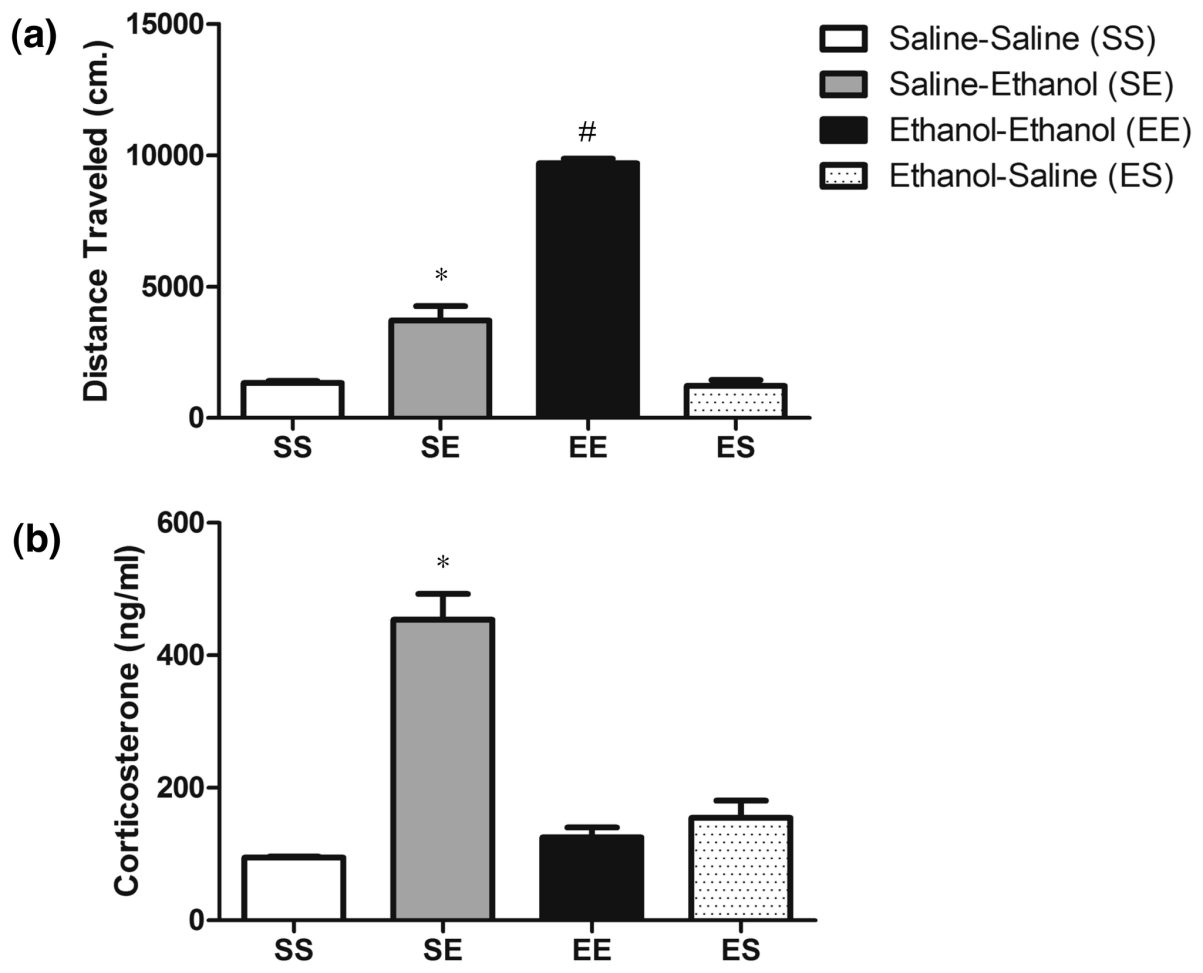
Serum corticosterone levels following ethanol sensitization. Behavioral sensitization followed by corticosterone quantification. Panels show: (a) Acute and sensitized locomotor response (cm/10 min.) following saline (SS, ES) or ethanol (EE, SE) administration. * p < 0.05 versus SS and ES groups, # p < 0.05 versus SE, ES and SS groups; (b) Corticosterone levels 1 hour following acute and chronic ethanol administration. * p < 0.05 versus SS, EE, and ES groups.

*Sgk1* is a well-known glucocorticoid responsive gene and it is known that animals and human alcoholics show a blunted HPA axis while drinking and upon withdrawal. Because *Sgk1* levels were not regulated in animals following ethanol sensitization despite normal levels of *Nr3c1* expression, we hypothesized that corticosterone levels may be blunted in animals chronically treated with ethanol (EE animals). We sensitized animals to ethanol and collected blood one hour following behavioral testing to measure corticosterone levels across SS, SE, EE and Ethanol-Saline (ES) treated animals. In evaluating the behavioral response to ethanol, there was again evidence of strong locomotor sensitization. A one-way ANOVA showed an overall effect of treatment (*F*_3,15_= 159.67, *p* < 0.01) (Figure **4a**). Post-hoc analysis revealed that EE treated animals showed a significantly greater locomotor response compared to SS, SE, and ES treated animals. In addition, SE treated animals showed a greater locomotor response compared to SS and ES treated animals. In evaluating corticosterone levels at one hour after injections of SS, SE, EE and ES mice, a one-way ANOVA showed an overall significant effect of treatment (*F*_3,15_= 47.37, *p* < 0.01) (Figure **4b**). Post-hoc analysis revealed that SE animals showed greater corticosterone levels than SS, EE and ES indicating that corticosterone levels were blunted with chronic ethanol treatment.

### Effects of ADX on Sgk1 induction following ethanol administration

**Figure 5:**
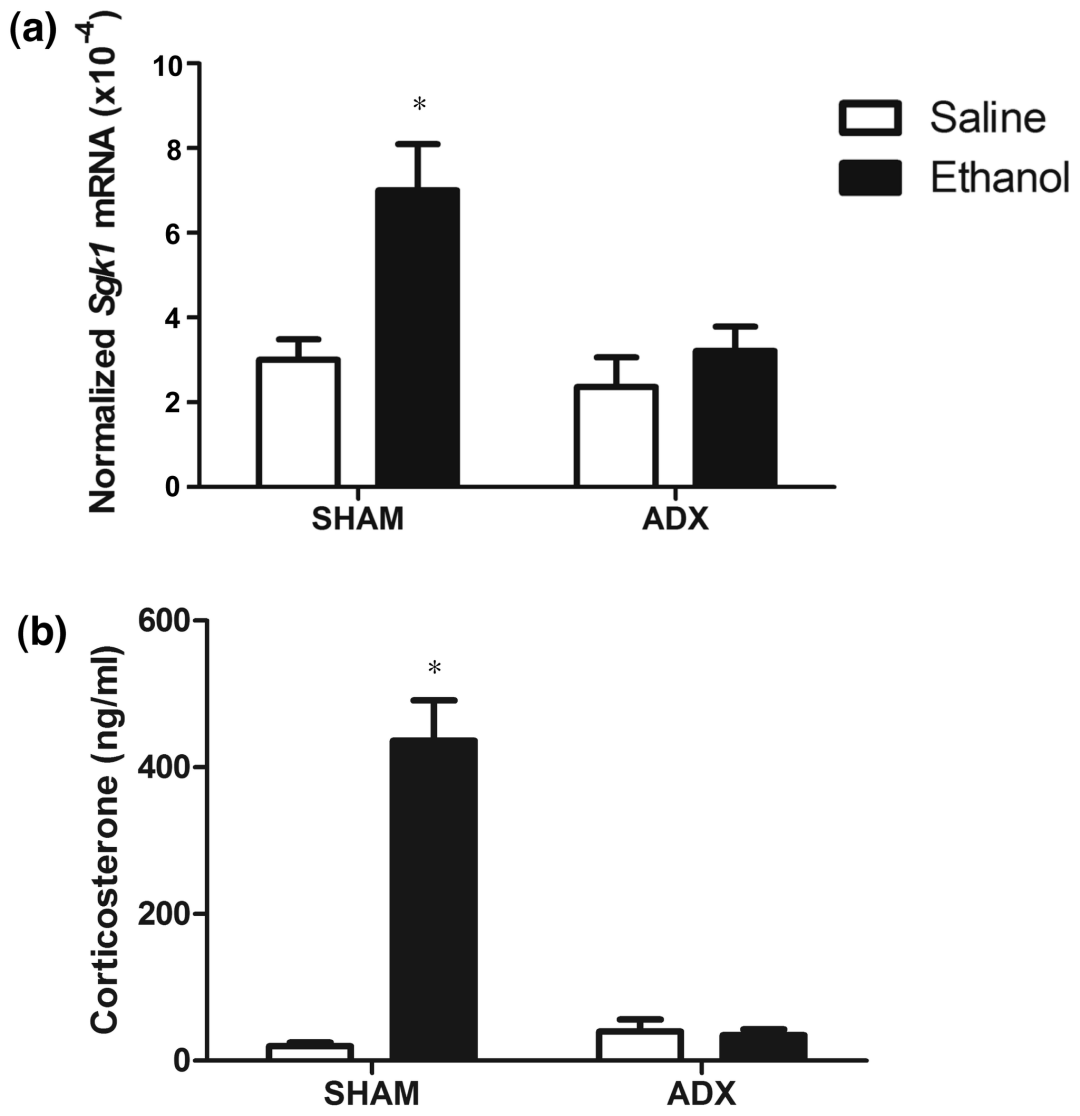
Effects of adrenalectomy on *Sgk1* induction following ethanol administration. (**a**) Q-rtPCR analysis of *Sgk1* in saline and ethanol treated SHAM versus ADX animals. * p < 0.05 versus saline treated SHAM animals and saline and ethanol treated ADX animals; (b) Corticosterone levels 1 hour following acute ethanol administration. * p < 0.05 versus saline treated SHAM animals and saline and ethanol treated ADX animals.

Because *Sgk1* was not induced in EE treated animals and the corticosterone response to ethanol was also blunted in these animals, we hypothesized that *Sgk1* would not be induced in ADX animals, animals lacking their adrenal glands, the organ responsible for corticosterone release and the final step in HPA axis activation. Q-rtPCR analysis of Sgk1 mRNA levels at 4 hours following ethanol (4 g/kg i.p.) showed an overall effect (two-way ANOVA, phenotype x treatment) of phenotype (*F*_1,22_= 7.16, *p* < 0.05), treatment (*F*_1,22_= 11.05, *p* < 0.01) and a significant (phenotype x treatment) interaction (*F*_1,22_= 5.31, *p* < 0.05) (Figure **5a**). Post-hoc analysis of phenotype indicated *Sgk1* levels were significantly increased in ethanol versus saline treated Sham animals, but not ADX animals. Post-hoc analysis of treatment indicated significant differences between ethanol, but not saline, treated Sham versus ADX mice indicating that *Sgk1* levels were blunted in ADX mice following ethanol administration. We also evaluated *Sgk1.1* (Figure **S3**) and found no differences between *Sgk1.1* levels in ADX versus Sham treated or saline versus ethanol treated animals.

To ensure that animals were, in fact, adrenalectomized, we evaluated corticosterone levels in ADX vs. SHAM animals. A two-way ANOVA (phenotype x treatment) indicated an overall effect of phenotype (*F*_1,14_= 52.22, *p* < 0.05), treatment (*F*_1,14_= 60.83, *p* < 0.01) and a significant (phenotype x treatment) interaction (*F*_1,14_= 63.91, *p* < 0.05). Post hoc analysis revealed that corticosterone levels were significantly greater in ethanol treated SHAM animals versus saline treated SHAM animals, saline treated ADX animals and ethanol treated ADX animals (Figure **5b**). Therefore, ADX mice did not experience increases in corticosterone following ethanol administration and the induction of *Sgk1* in D2 mice may be due to HPA axis activation and glucocorticoid signaling following acute ethanol administration.

### Chromatin Immunoprecipitation (ChIP) Assay

**Figure 6:**
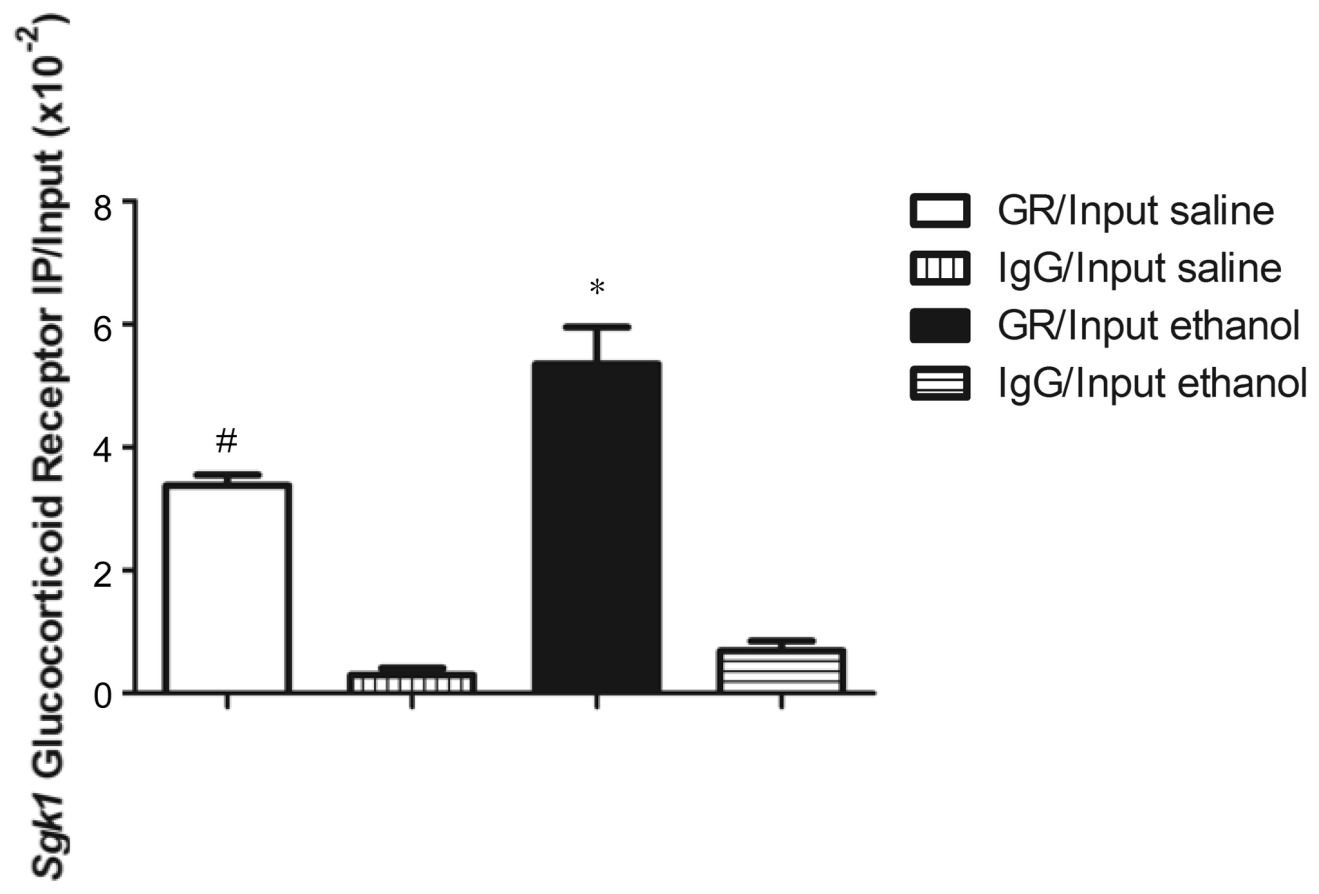
Chromatin immunoprecipitation (ChIP) quantification of glucocorticoid receptor (GR) bound to *Sgk1* promoter region. DBA2/J mice were treated with saline or ethanol (4 g/kg) by i.p. injection and chromatin isolated 1 hour later from a frontal pole dissection. *Sgk1* promoter DNA was quantified by PCR following immunoprecipitation by control IgG or GR antibody. * p < 0.05 versus GR saline treated samples and IgG saline and ethanol treated samples, # p < 0.05 versus IgG saline and ethanol treated samples.

To test whether *Sgk1* induction following ethanol administration was due to glucocorticoid receptor (GR) binding to the glucocorticoid response element (GRE) in the *Sgk1* promoter, we performed a chromatin immunoprecipitation (ChIP) assay in which we isolated chromatin from ethanol and saline treated animals and conducted immunoprecipitation studies using an anti-GR antibody and a control anti-IgG antibody. A two-way ANOVA (antibody x treatment) showed an overall effect of antibody (F_1,8_= 140.40, p < 0.01), treatment (F_1,8_= 13.13, p < 0.01) and a significant (treatment x antibody) interaction (F_1,8_= 5.85, p < 0.05). Post-hoc analysis revealed that looking at samples in which GR immunoprecipitations were performed, the level of *Sgk1* promoter region bound to the GR in ethanol treated samples was significantly greater than that bound in saline treated samples. In samples in which IgG immunoprecipitations were performed, there were no significant differences between *Sgk1* promotor region bound to the IgG antibody in ethanol versus saline treated samples. Additionally, looking at antibody effects within saline treated animals, there was a significantly greater amount of Sgk1 promoter region bound to the GR antibody versus the IgG antibody. Looking at antibody effects within ethanol treated animals, there was a significantly greater amount of *Sgk1* promoter region bound to the GR antibody versus the IgG antibody (Figure **6**). Therefore, we surmise that *Sgk1* induction in PFC following acute ethanol administration is due to activation of the HPA axis and the subsequent binding of corticosterone-activated GR to the GRE of the *Sgk1* promoter.

### Acute ethanol regulation of SGK1 protein abundance, phosphorylation and functional activity

**Figure 7:**
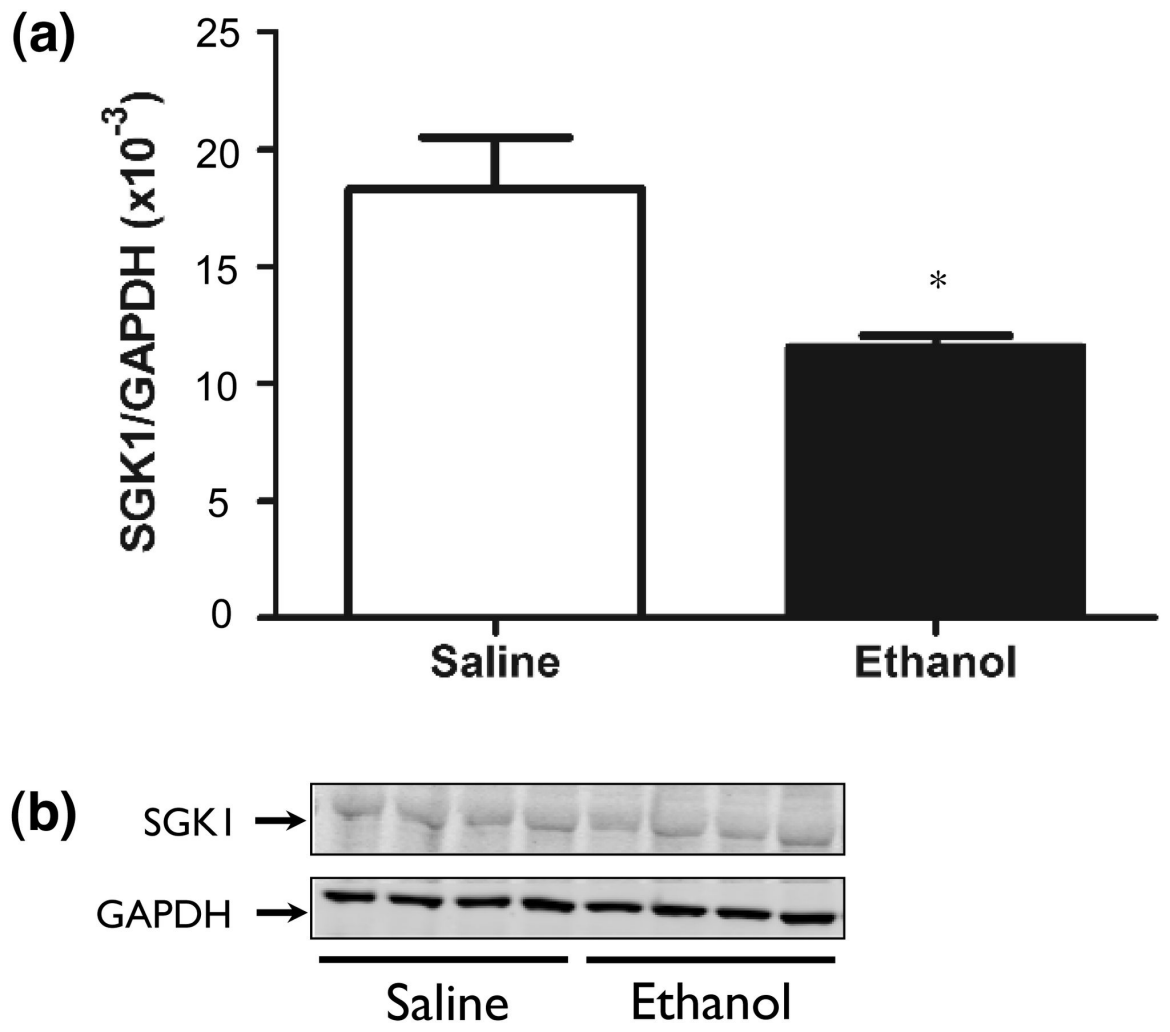
Total SGK1 protein following ethanol administration. Western blot analysis of SGK1 8 hours following ethanol (4g/kg) or saline treatment. Panels show: (a) Quantification of SGK1, (b) Representative Western blot. * p < 0.05 versus saline treated animals.

In addition to the transcriptional regulation studied above, SGK1 is prominently regulated by multiple sites of protein phosphorylation [22,29,30]. In particular, phosphorylation at sites S422 and T256 are known to activate the kinase [30–32]. We therefore performed a detailed analysis on ethanol regulation of SGK1 protein abundance and phosphorylation. A time course (0.25-24 hours) Western blot analysis was performed for SGK1 protein and phospho S422 (pS422) and T256 (pT256) levels following ethanol (4 g/kg) versus saline treatment. Given the prior documented increases in *Sgk1* mRNA, we expected that we would find increased levels of total SGK1 protein. Surprisingly, we found significant decreases in total SGK1 protein in ethanol versus saline treated animals 8 hours following ethanol administration (student’s t-test, *p* < 0.05) (Figure **7a-b**). At all other time points studied (15 min, 1 h, 2 h, 4 h and 24 h), there were no significant differences in SGK1 levels between saline and ethanol treated animals (Figure **S4a–f**). We also compared basal SGK1 levels to SGK1 levels 15 min and 8 h following saline administration, time points where we found alterations in SGK1 protein levels (8 h) or phosphorylated, active SGK1 (15 min). We found no differences in SGK1 levels at any of these time points (Figure **S5a-b**).

**Figure 8:**
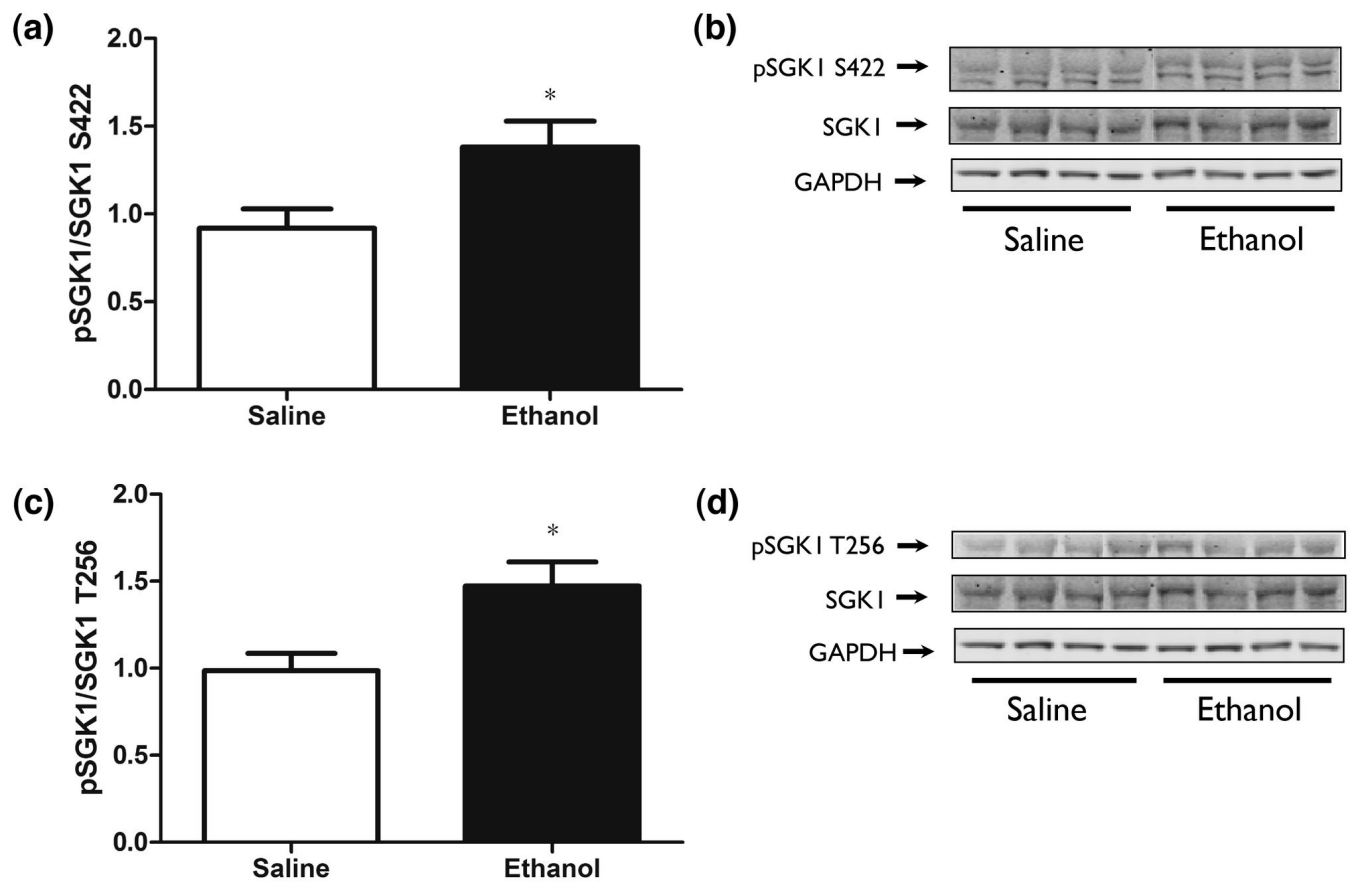
pSGK1 following acute saline and ethanol administration. Western blot analysis of pS422 SGK1 and pT256 SGK1 at 15 minutes following ethanol (4g/kg) or saline treatment. Panels show: (a) Quantification of pS422 SGK1, (b) Representative Western blot, c) Quantification of pT256 SGK1, and (d) Representative Western blot. * p < 0.05 versus saline treated animals.

Studying the same time course described above for Western blot analysis of total SGK1, we found a significant increase in SGK1 pS422 and pT256 15 min. following ethanol treatment (student’s t-test, *p* < 0.05) (Figure **8a–d**). However there were no differences between saline- and ethanol-treated animals 1-24 hours following drug treatment (Figure **S6a–f**). This suggests a rapid activation of SGK1 by phosphorylation at both S422 and T256 as early as 15 minutes following ethanol treatment. We also compared basal SGK1 pS422 levels to SGK1 pS422 levels 15 min and 8 h following saline administration, time points where we found alterations in SGK1 protein levels (8 h) or phosphorylated, active SGK1 (15 min). We found no differences in SGK1 pS422 levels at any of these time points (Figure **S7a-b**).

**Figure 9:**
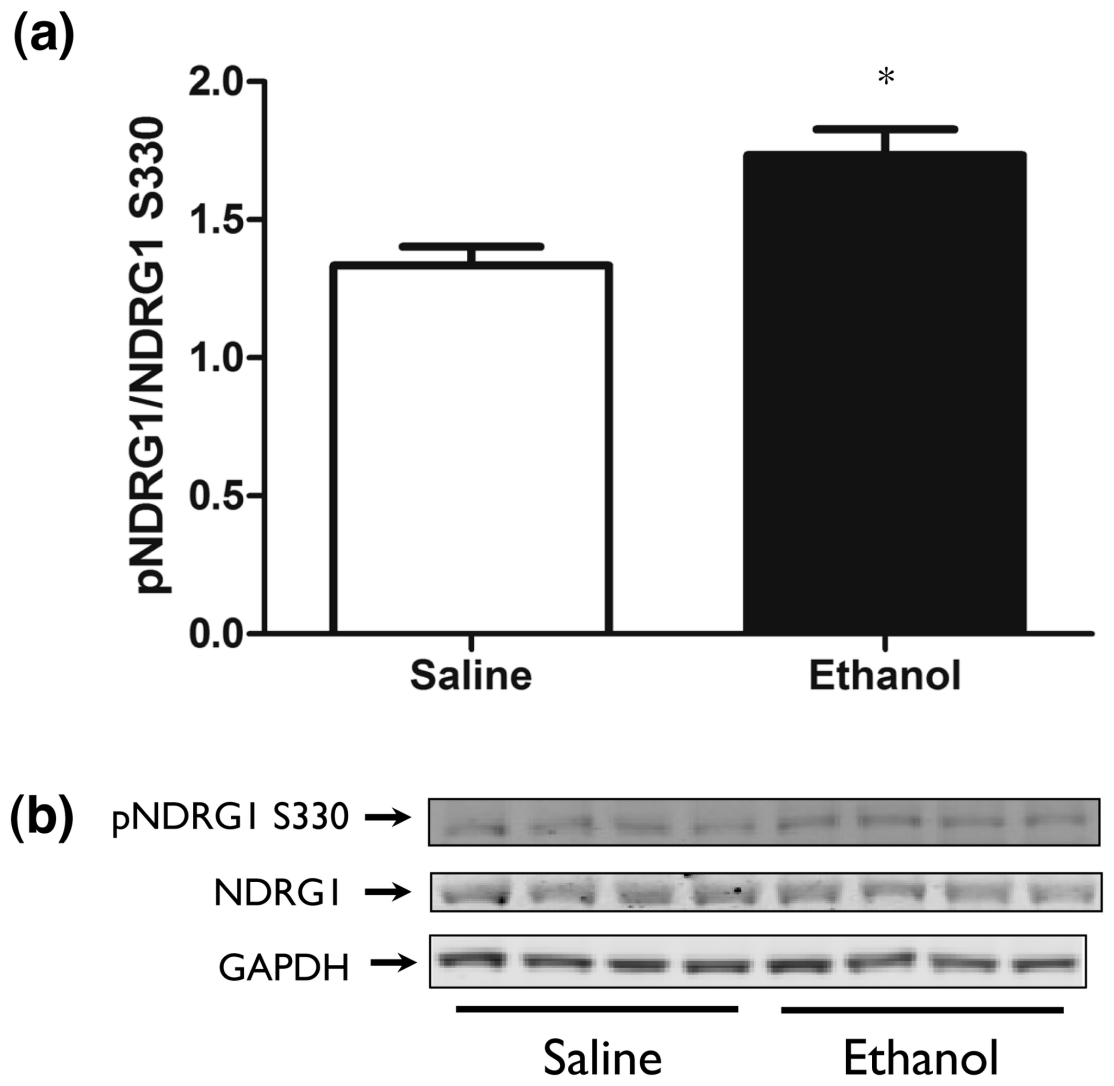
NDRG1 phospho S330 following ethanol administration. Western blot analysis of pS330 NDRG1 15 minutes following ethanol (4g/kg) or saline treatment. Panels show: (a) Quantification of pS330 NDRG1, (b) Representative Western blot. * p < 0.05 versus saline treated animals.

Phosphorylation events should increase SGK1 kinase activity. To support activation of SGK1, we studied phosphorylation of NDRG1, a well-characterized downstream target of phosphorylated SGK1 [33]. Western blot analysis confirmed that ethanol treatment produced significant increases in NDRG1-phospho S330 versus saline-treated animals in PFC at 15 minutes following ethanol addition (student’s t-test, *p* < 0.05) (Figure **9a-b**).

### Chronic ethanol regulation of SGK1 phosphorylation

**Figure 10:**
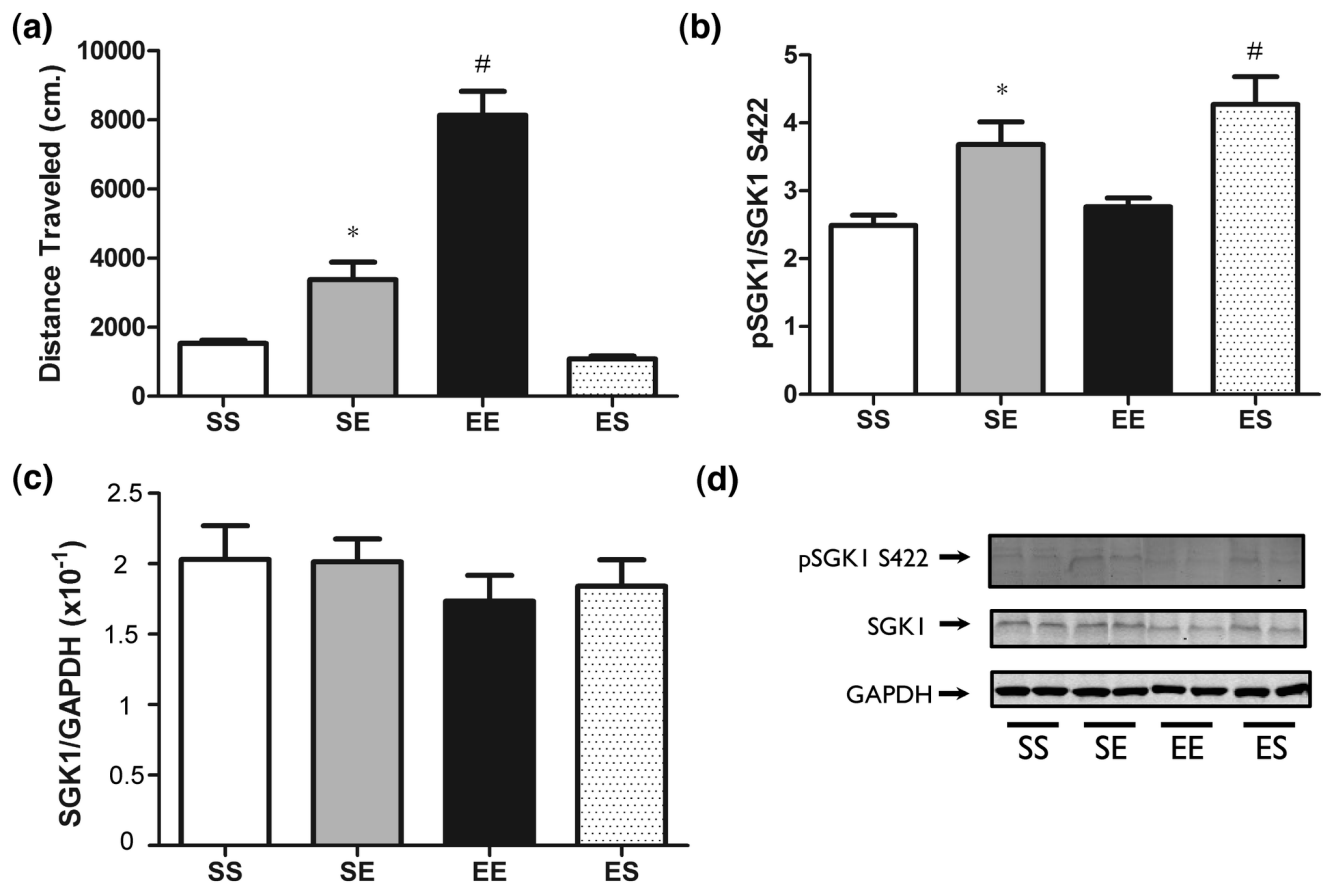
pSGK1 following chronic saline and ethanol administration. Western blot analysis of pS422 SGK1 15 minutes following chronic saline (SS), acute ethanol (2g/kg) (SE), chronic ethanol (EE) or acute saline (ES) treatment. Panels show: (a) Acute and sensitized locomotor response (cm/10 min.) following saline (SS, ES) or ethanol (EE, SE) administration. * p < 0.05 versus SS and ES groups, # p < 0.05 versus SE, ES and SS groups, (b) Quantification of pS422 SGK1, * p < 0.05 versus SS and EE groups, # p < 0.05 versus SS and EE groups, c) Quantification of SGK1, and (d) Representative Western blot.

We have shown that *Sgk1*, *Fkbp5* and possibly other glucocorticoid responsive genes were no longer regulated following chronic ethanol administration (Figure **3a–d**). However, due to the disparate responses of Sgk1 mRNA and protein levels (Figure **8a-b**) with acute ethanol, we hypothesized that SGK1 protein levels or phosphorylation might show compensatory changes to the blunted HPA axis. We therefore evaluated SGK1 pS422 levels following chronic ethanol sensitization studies. There again was overall effect of treatment on locomotor activity (one-way ANOVA, *F*_3,27_= 45.87 *p* < 0.01) (Figure **10a**). Post-hoc analysis revealed that ethanol-ethanol (EE) treated animals showed significantly greater locomotor activity compared to saline-ethanol (SE), saline-saline (SS) and ethanol-saline (ES) treated animals. In addition, SE treated animals showed greater locomotor activity than SS and ES treated animals. In evaluating SGK1 pS422 levels in these animals, there was an overall significant effect of treatment on SGK1 pS422 levels (one-way ANOVA, *F*_3,21_= 8.75, *p* < 0.01) (Figure **10b,d**). Post-hoc analysis showed that SGK1 pS422 levels were significantly increased in animals treated acutely with ethanol compared to SS and EE treated animals. Interestingly, SGK1 pS422 levels were also significantly elevated in ES treated animals compared to SS and EE treated animals. SGK1 pS422 did not differ between SS and EE treated animals. Total SGK1 levels did not differ between SS, SE, EE and ES treated animals in any group 15 minutes following acute ethanol administration (Figure **10c-d**).

### SGK1 and NDRG1 Basal Expression in Neurons and Oligodendrocytes

**Figure 11:**
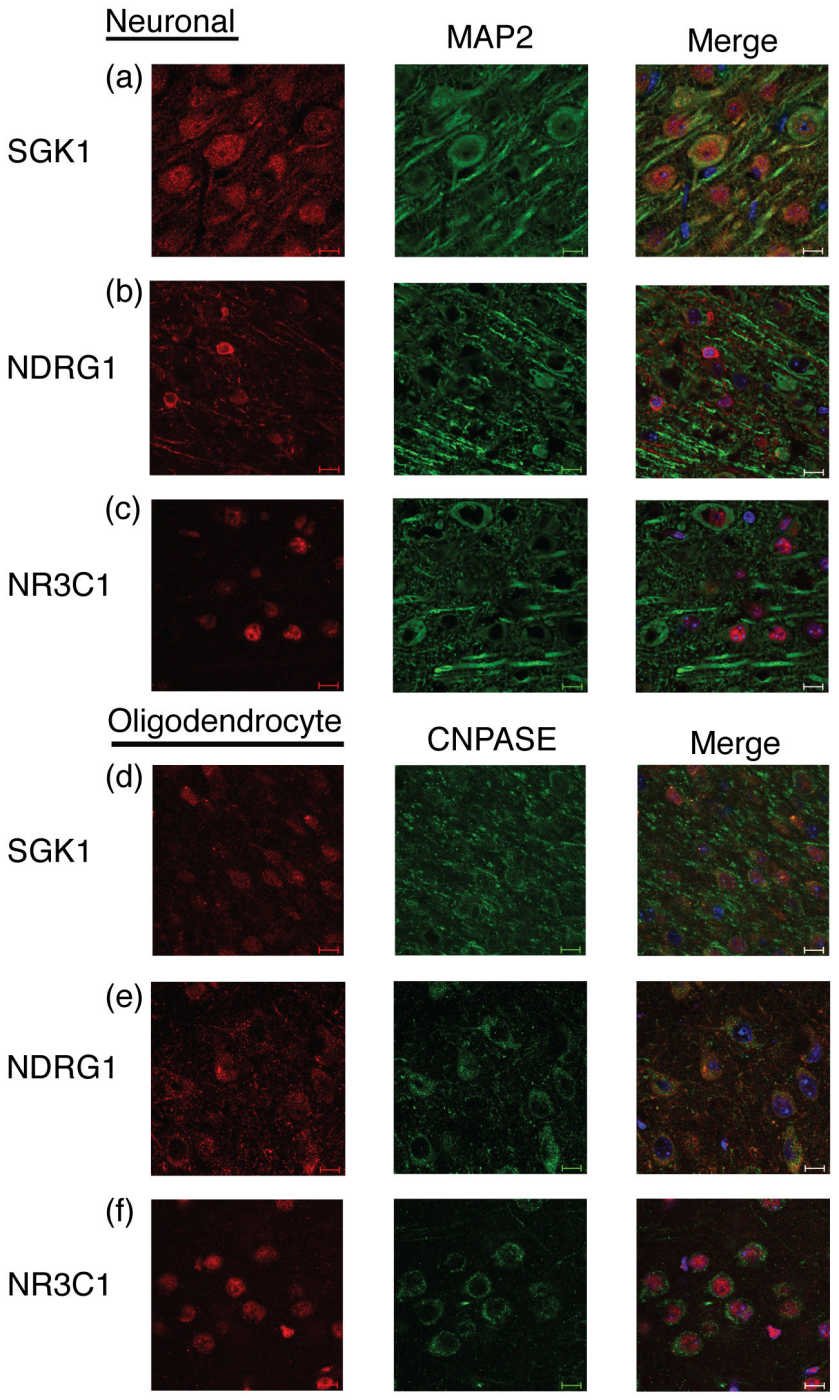
SGK1, NDRG1, and NR3C1 basal expression patterns in neurons and oligodendrocytes in the PFC of D2 mice. NDRG1, NR3C1 and SGK1 were co-localized with the neuronal marker MAP2 and the oligodendrocyte marker CNPase. DAPI staining was used as a nuclear marker (right columns). Panels show (from left to right): (a) SGK1 staining, MAP2 staining, co-localization; (b) NDRG1 staining, MAP2 staining, co-localization; (c) NR3C1 staining, MAP2 staining, co-localization; (d) SGK1 staining, CNPASE staining, co-localization; (e) NDRG1 staining, CNPASE staining, co-localization; (f) NR3C1 staining, CNPASE staining, co-localization. Scale bar equals 50 µm.

The studies on NDRG1 phosphorylation (Figure 9a-9b) suggested that Sgk1 activation by ethanol might be occurring in oligodendrocytes since NDRG1 is known to predominantly expressed in that cell type within the central nervous system. We performed immunohistochemistry to further decipher the cell type(s) in which SGK1 may respond to ethanol and transduce downstream signaling. We performed double labeling experiments using neuronal (MAP2) and oligodendrocyte (CNPase) markers in addition to NR3C1, NDRG1 and SGK1 labeling to determine the cell types where NR3C1, SGK1 and NDRG1 are expressed basally in the PFC of D2 mice. We found that SGK1 and NR3C1 are expressed in both neurons and oligodendrocytes in the PFC of D2 mice (Figure **11a,c,d,f**). Whereas, NDRG1 is only expressed in oligodendrocytes (Figure **11b,e**). Neurons, cells positive for MAP2, were also positive for SGK1 (Figure **11a**) and NR3C1 (Figure **11c**), but not NDRG1 (Figure **11b**) at least as detectable by immunofluorescence. Oligodendrocytes, cells positive for CNPase, were also positive for SGK1 (Figure **11d**), NDRG1 (Figure **11e**) and NR3C1 (Figure **11f**). These results suggest that SGK1 could regulate acute responses to ethanol in both neurons and oligodendrocytes through glucocorticoid signaling mechanisms. Additionally, NDRG1 signaling might have a role as a downstream mediator of SGK1 responses to acute ethanol in oligodendrocytes.

## Discussion

Prior studies in our laboratory and others suggest that glucocorticoid signaling might play an important role in both behavioral and brain gene expression responses to acute ethanol [12,15,17]. Using genomic studies we previously identified a significant over-representation of glucocorticoid-responsive genes, including *Sgk1*, responding to acute ethanol in PFC [12]. More recent studies from our laboratory showed that inhibition of glucocorticoid signaling by adrenalectomy or the glucocorticoid antagonist RU486 both impaired acute ethanol-induced locomotor activation, suggesting that glucocorticoid signaling may play a role in this behavioral response [15]. Further, ADX altered the basal expression of genes in PFC that included a significant number of previously identified ethanol responsive genes [15]. Although there are other conflicting reports about the role of glucocorticoids in ethanol behaviors, recent studies elegantly document that blockade of glucocorticoid signaling severely impairs escalated ethanol consumption in an animal model of progressive ethanol intake [34]. Furthermore, HPA axis dysregulation exists in alcohol dependent patients and individuals with a familial history of alcoholism [35,36]. Thus, disrupted HPA axis/glucocorticoid signaling may contribute to the risk for development of alcohol dependence. In this report, we have provided mechanistic studies confirming that ethanol modulates brain (PFC) gene expression in part through glucocorticoid signaling and that alterations in this gene expression regulatory loop occur with chronic exposure.

Prior studies in our laboratory and others identified the glucocorticoid-regulated gene Sgk1 as an ethanol-responsive gene [12,13,17]. Other investigators have described *Sgk1* regulation following ethanol administration in whole brain [13] and in striatum [17], but not in the PFC—a region known to be important in glucocorticoid signaling [37]. Here we performed a rigorous analysis of *Sgk1* regulation in PFC by ethanol. Although *Sgk1* is the predominant transcript from the gene, an alternative promoter produces the transcript *Sgk1.1* that codes for a more stable form of the SGK protein (Figure 1) [25]. However, ethanol only regulated expression of *Sgk1* (Figure 2), which contains a glucocorticoid response element (GRE) in its promoter [19,25]. We show for the first time, that ethanol regulation of *Sgk1* occurred via evoked glucocorticoid signaling since adrenalectomy blocked ethanol induction of *Sgk1* expression (Figure 5) and ethanol increased occupancy of glucocorticoid receptor binding to the *Sgk1* promoter in ChIP assays (Figure 6). Ethanol regulation of *Sgk1* transcription is thus part of a HPA-PFC regulatory loop that could have an important role in modulating the acute cellular response to ethanol. The PFC is known to have high concentrations of glucocorticoid receptors and to modulate HPA axis activity [37]. The PFC is also part of the mesolimbocortical dopamine pathway that is involved in the rewarding properties of drugs of abuse and is thought to contribute to the known interaction between stress and ethanol consumption [38].

Ethanol regulation of *Sgk1* versus *Sgk1.1* may have functional implications since the two isoforms have different downstream targets. *Sgk1* stimulates K^+^ channel activity and regulates the function and availability of the epithelial sodium channel (ENaC) [20,39,40]. *Sgk1.1* is a brain specific isoform of *Sgk1* that has been shown to modulate the function of the acid-sensing ion channel-1, the δENaC and M-current [25,41,42].

Following chronic ethanol treatment to produce locomotor sensitization, *Sgk1* and serum corticosterone levels no longer responded to ethanol treatment (Figures 3b, 4b). This provided further evidence linking glucocorticoid signaling and ethanol regulation of *Sgk1*. Furthermore, by dampening of the HPA response to ethanol, a network of glucocorticoid-responsive genes in PFC including Sgk1 and Fkbp5 have undergone an adaptive response to chronic ethanol exposure. Although additional future studies are needed to fully characterize the response, our studies on SGK1 protein and phosphorylation suggest the possibility that basal SGK1 activity might be increased following chronic ethanol treatments during locomotor sensitization, perhaps in compensation to the down-regulated HPA axis responsiveness or other direct responses to chronic ethanol.

While the studies here were not designed to prove or disprove a role for Sgk1 in ethanol sensitization, our findings do suggest testable hypotheses for future work. The dampening of an acute ethanol and glucocorticoid-responsive gene network (including Sgk1 and Fkbp5) in PFC could have important implications for behavioral responses to chronic ethanol and stress. Moreover, our results showing that basal SGK1 phosphorylation is increased following sensitization (Figure 10), might directly affect ethanol-responsive locomotor activation. Ongoing studies including more detailed evaluation of post-translational changes in SGK1 following sensitization and direct alteration of SGK1 levels or phosphorylation state are necessary to definitively prove a link between Sgk1 and behavioral responses to acute or chronic ethanol.

Other investigators have shown a similar trend in corticosterone levels following chronic ethanol administration; as corticosterone levels are blunted in male rats administered ethanol following chronic operant self-administration, chronic intragastric ethanol administration and rats and mice following chronic i.p. injection [9,11,43,44]. However, this is the first work relating HPA axis dampening (or “tolerance”) to changes in PFC gene expression. Although other explanations exist for lack of ethanol-responsiveness in *Sgk1* following chronic ethanol treatment, such as decreased glucocorticoid receptor (GR) expression in PFC, the dampened corticosterone response to ethanol (Figure 4) and ChIP results showing increased GR binding to the *Sgk1* promoter with acute ethanol (Figure 6) are strong evidence linking the diminished *Sgk1* response to corticosterone levels. There was some GR binding to the GRE in the *Sgk1* promoter in saline treated animals in our ChIP studies. Although we habituated animals to injections for two days prior to performing our experiments, this result may reflect the stress of the injections or other environmental factors on our experimental test day. Diminished GR (*Nr3c1*) expression has been seen in the rat PFC following chronic ethanol exposure [34], but our Q-rtPCR results showed no significant changes in *Nr3c1* mRNA levels in the PFC of SS, SE vs EE treated animals (Figure 3d.). Although we did not measure blood ethanol concentrations (BECs) in our studies, prior studies with chronic ethanol showed corticosterone levels were blunted despite elevated BECs [9,43]. Of possible mechanistic importance, Roberts et al. reported that co-treatment with the glucocorticoid antagonist RU486 blocked the decrement in corticosterone during repeated ethanol treatment for sensitization [11]. RU486 also partially blocked ethanol locomotor sensitization in those experiments, although our prior work has not replicated this finding possibly due to methodological differences [15].

These studies also identified complex ethanol actions on SGK1 expression at the level of protein expression and post-translational modification (Figures 7-8). The activation of SGK1 is triggered first by the phosphorylation of S422 lying within the C-terminal hydrophobic motif of SGK1 followed by the phosphorylation of a threonine residue within the T-loop of the kinase domain [29,30,32]. Mammalian target of rapamycin (mTORC) was recently identified as the kinase that phosphorylates SGK1 at Ser422, but there is debate as to whether mTOR complex 1 or 2 is responsible for this phosphorylation [45,46]. We found that phosphorylation of SGK1 at S422 and T256 is transiently increased 15 minutes following ethanol administration (Figure 8a. -d.) but that total SGK1 protein abundance is significantly decreased as early as 6 hours (data not shown) and as late as 8 hours (Figure 7a. -b, Figure S4) following ethanol administration. This is somewhat paradoxical since *Sgk1* mRNA abundance is increased at 2-4 hours after ethanol treatment (Figure 2a). The dissociation of Sgk1 mRNA, total protein and phosphorylation state reinforces the need for such concurrent studies for understanding the mechanisms of ethanol action on such dynamically regulated target as Sgk1.

The transient increase in SGK1 Ser422 phosphorylation appears to be functional in that there is concomitant phosphorylation of the known SGK1 substrate protein, NDRG1 (Figure 9a-b). NDRG1 is a well-known SGK1-specific substrate minimally phosphorylated by other kinases and thought to be predominantly expressed in oligodendrocytes in the central nervous system [33,45,47]. Miyata et al. showed that a chronic stress paradigm that elevated plasma corticosterone levels led to activation of the PDK1, SGK1, and NDRG1 pathway in mouse oligodendrocytes [48]. There have been additional alternative findings in the literature as to the cell type(s) where SGK1 and NDRG1 mediate their effects. Okuda et al. identified NDRG1 as mainly localized in oligodendrocytes in mouse cerebrum [49]. Works examining SGK1 location in the human brain of aged Alzheimer’s versus control patients documented SGK1 location to be primarily neuronal [47]. However, other work has found neurons, oligodendrocytes, and microglial cells, but not astrocytes, were positive for SGK1 in the rat [50]. These alternate findings can be partially explained by species and methodological differences.

Our immunofluorescence studies documented overlapping basal expression patters for NDRG1, SGK1 and NR3C1 (GR). These studies do not document the precise cell type responsible for the mRNA and protein changes identified in this work, However, we did document that NDRG1, SGK1 and GR are all expressed in cells co-labeling with the oligodendrocyte marker CNPase (Figure 11d–f). The increased NDRG1 phosphorylation after acute ethanol suggests that SGK1 activation by ethanol is occurring, at least in part, in oligodendrocytes. However, since GR and SGK1 are also co-expressed in cells labeling with the neuronal marker MAP2 (Figure 11a–c), there is also the possibility that ethanol regulates Sgk1 expression in neurons as well. Future quantitative immunohistochemistry or in situ hybridization studies will be needed to more directly determine the cellular site of ethanol regulation of Sgk1.

This study is the first description of this complex ethanol regulation of *Sgk1* mRNA, protein and phosphorylation. Similar to our findings here, Piechota et al. showed *Sgk1* mRNA is significantly increased 2 hours following ethanol, morphine, heroin and methamphetamine administration and that SGK1 protein is significantly decreased 4 hours following morphine administration in the striatum. But those investigators did not study SGK1 phosphorylation or protein regulation following ethanol administration [17]. It is possible that ethanol triggers a complex wave of signaling events leading to: 1) SGK1 activation by phosphorylation with subsequent phosphorylation of NDRG1 and other targets; 2) increased *Sgk1* transcription by HPA axis activation and glucocorticoid action; and 3) compensatory SGK1 protein degradation. Of note, Miyata et al. showed that a chronic stress paradigm that elevated plasma corticosterone levels similar to those found in depressed individuals led to activation of the phosphatidylinositol 3-kinase (PI3K)-3-phosphoinositide-dependent protein kinase (PDK1), SGK1, and NDRG1 pathway with increases in both *Sgk1* mRNA and SGK1 phosphorylation [48]. Thus, it is possible that HPA axis activation is causal in both SGK1 activation and increased transcription of the *Sgk1* gene. Our findings of increased SGK1 phosphorylation in sensitized animals, however, would seem to dissociate HPA axis-derived glucocorticoid signaling from the phosphorylation state of Sgk1.

Overall our findings support clinical reports showing stress hypo-responsiveness in human alcoholics and provide evidence for how HPA axis tolerance can alter ethanol responses in brain stress/reward related regions such as PFC [51–53]. While changes occurring at the endocrine level are characterized, less defined are molecular changes in brain stress/reward related regions that are mediated by HPA axis tolerance. Our work here clearly indicates that future studies are warranted to further characterize chronic ethanol regulation of PFC expression networks, and determine whether alterations in HPA-regulated genes such as SGK1 are mechanistically linked to behavioral adaptations seen with chronic ethanol exposure.

## Supporting Information

Figure S1 **Sgk1 levels in the PFC of D2 mice basally (a 0 hour time point) and 2, 4 and 8 hours following saline injections.**
Saline injections did not significantly alter Sgk1 levels at any time point compared to basal Sgk1 levels.
(TIF)

Figure S2 ***Sgk1.1* levels following ethanol sensitization.**
*Sgk1.1* levels in SS, SE and EE mice.
(TIF)

Figure S3 **Q-rtPCR analysis of *Sgk1*.*1*.**
*Sgk1.1* in saline and ethanol treated SHAM versus ADX animals.
(TIF)

Figure S4 **Time Course Western blot analysis of total SGK1.**
SGK1 was significantly decreased 8 hours following ethanol versus saline administration (e). There were no significant changes in SGK1 levels at any other time point (a–d, f). * p < 0.05 versus saline treated animals.
(TIF)

Figure S5 **Basal versus saline treated SGK1 levels.**
There were no differences in SGK1 levels basally, 15 minutes following saline injection or 8 hours following saline injection. Panels show: (a) Quantification of SGK1, (b) Representative Western blot.
(TIF)

Figure S6 **Time Course Western blot analysis of pSGK1 S422.**
pSGK1 S422 was significantly increased 15 minutes following ethanol versus saline administration (a). There were no significant changes in pSGK1 S422 levels at any other time point (b–f). * p < 0.05 versus saline treated animals.
(TIF)

Figure S7 **Basal versus saline treated SGK1 pS422 levels.**
There were no differences in SGK1 pS422 levels basally, 15 minutes following saline injection or 8 hours following saline injection. Panels show: (a) Quantification of SGK1 pS422, (b) Representative Western blot.
(TIF)
