# Supplementary figures and images for: Ethanol Regulation of Serum Glucocorticoid Kinase 1 Expression in DBA2/J Mouse Prefrontal Cortex
Source: PLoS One. 2013 Aug 22;8(8):e72979. doi: 10.1371/journal.pone.0072979 (PMC3750005; doi:10.1371/journal.pone.0072979)

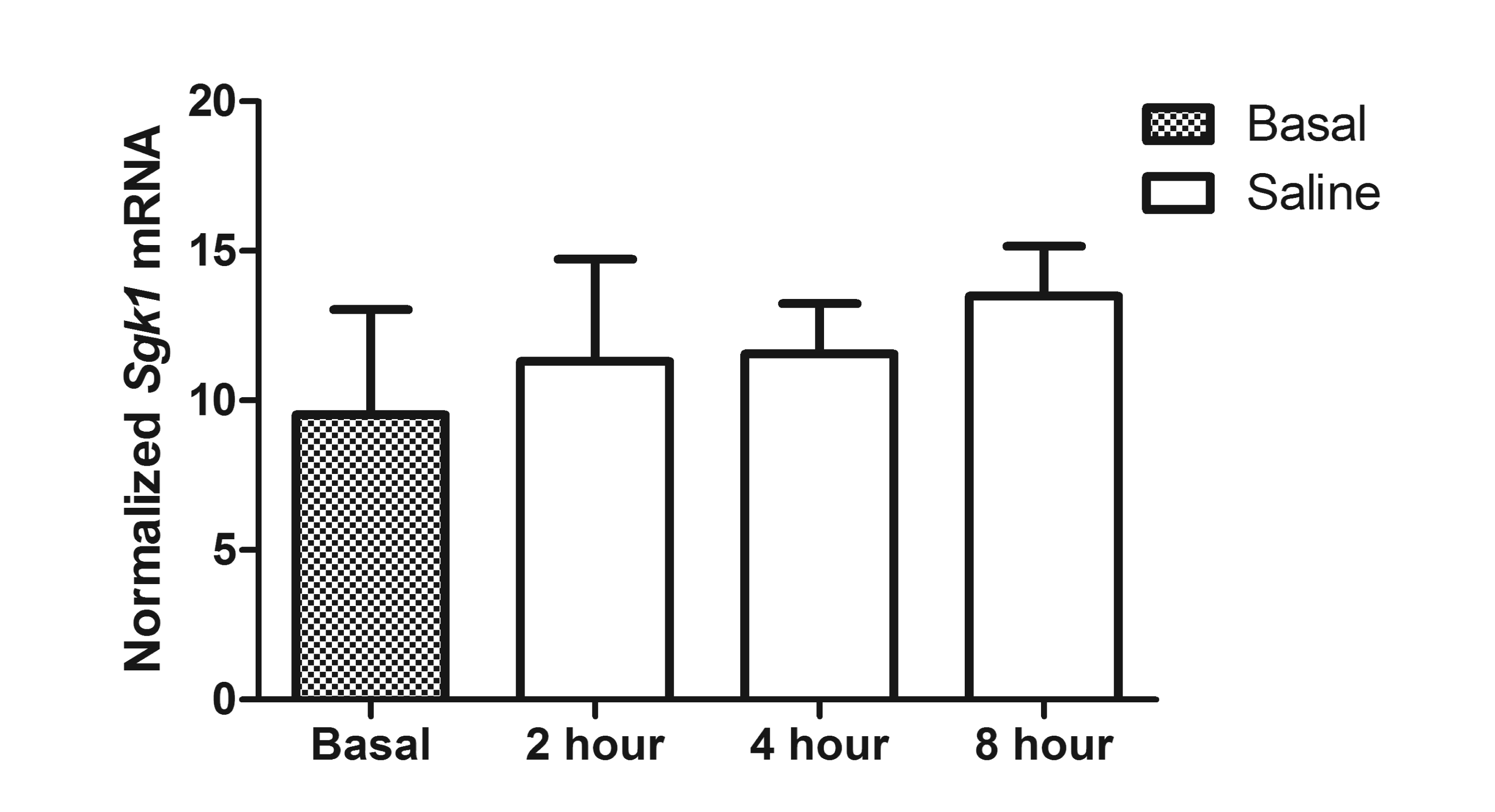

Supplement: Figure S1 — Sgk1 levels in the PFC of D2 mice basally (a 0 hour time point) and 2, 4 and 8 hours following saline injections. Saline injections did not significantly alter Sgk1 levels at any time point compared to basal Sgk1 levels. (TIF) [file pone.0072979.s001.tif]

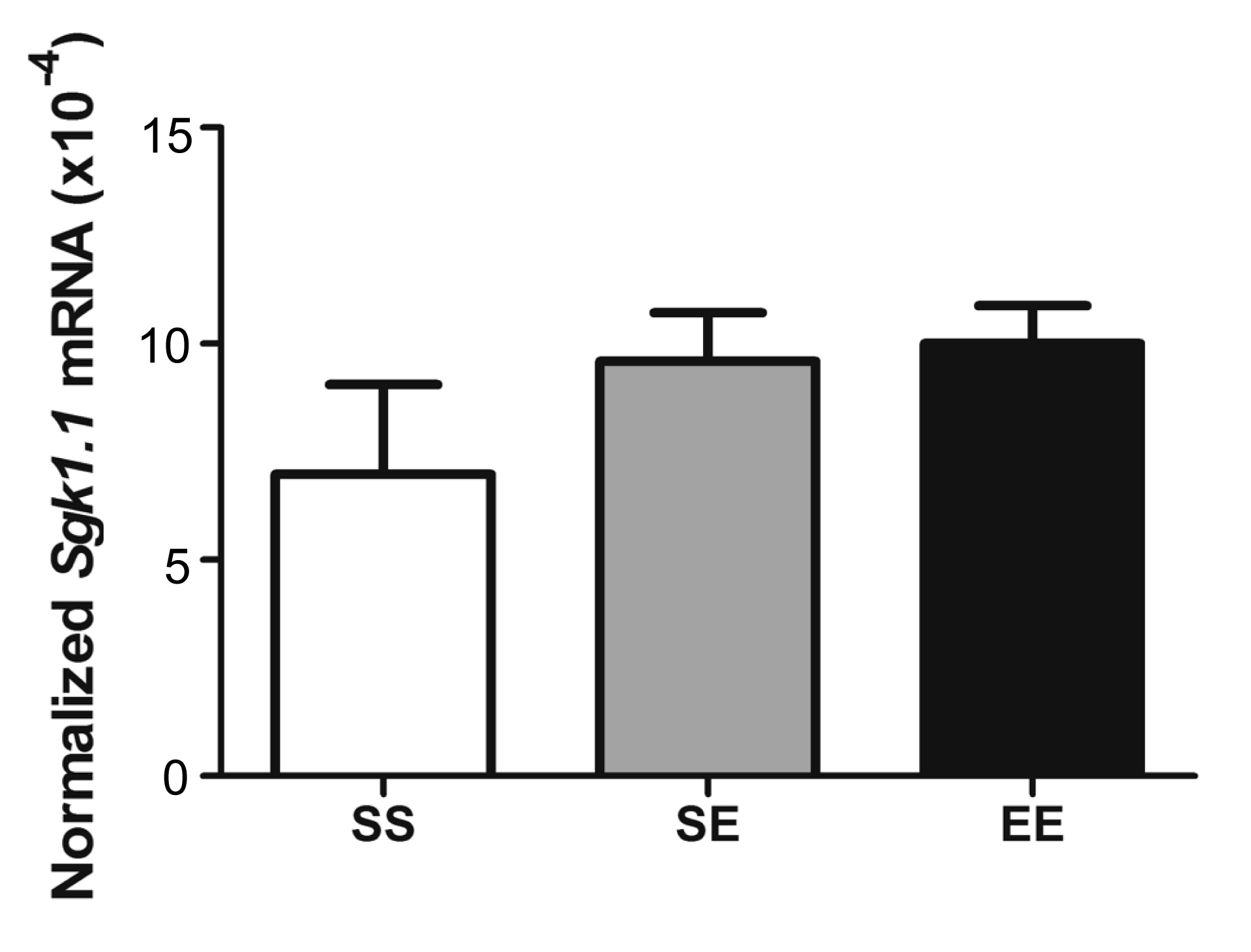

Supplement: Figure S2 — Sgk1.1 levels following ethanol sensitization. Sgk1.1 levels in SS, SE and EE mice. (TIF) [file pone.0072979.s002.tif]

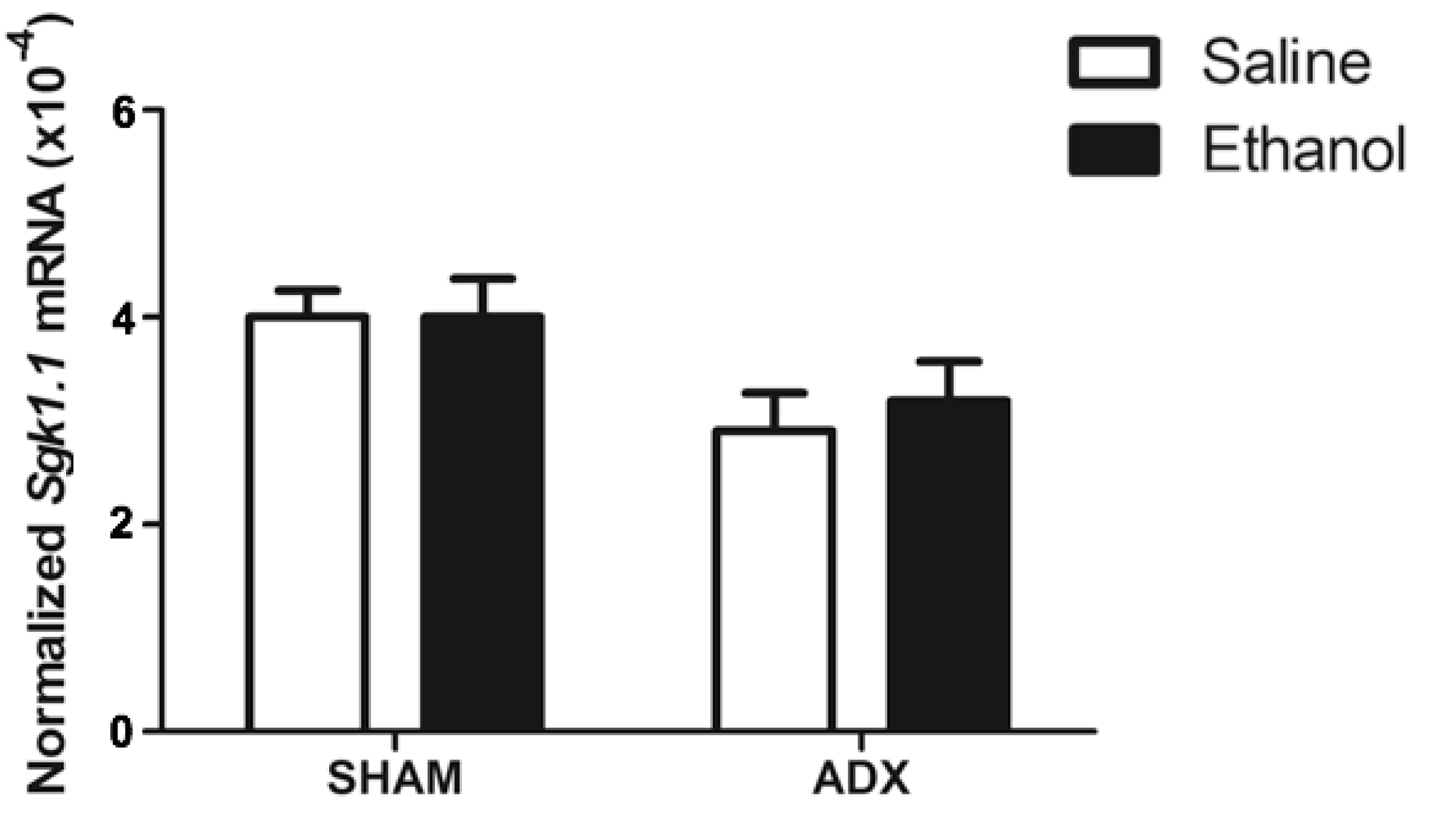

Supplement: Figure S3 — Q-rtPCR analysis of Sgk1.1. Sgk1.1 in saline and ethanol treated SHAM versus ADX animals. (TIF) [file pone.0072979.s003.tif]

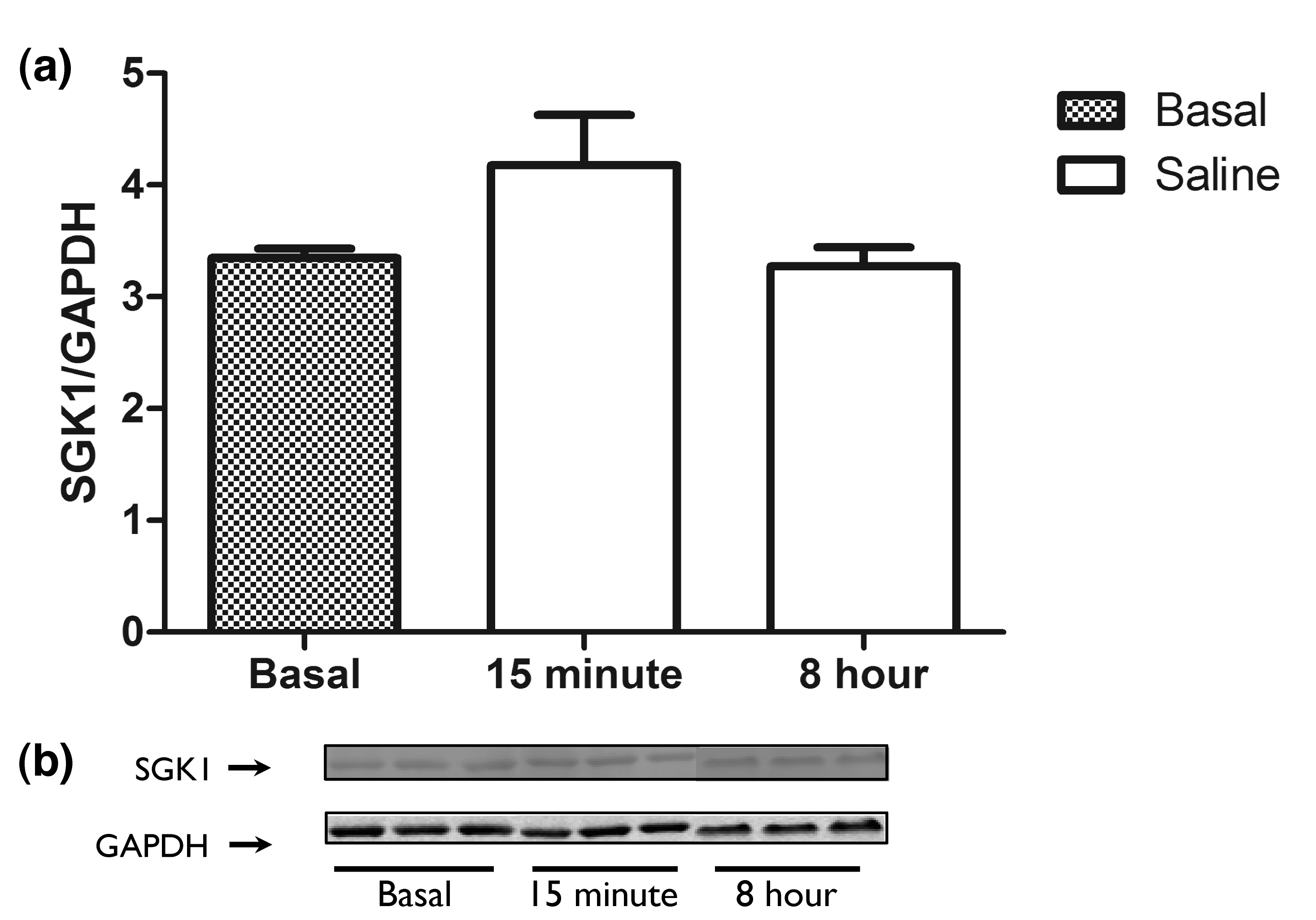

Supplement: Figure S4 — Time Course Western blot analysis of total SGK1. SGK1 was significantly decreased 8 hours following ethanol versus saline administration (e). There were no significant changes in SGK1 levels at any other time point (a–d, f). * p < 0.05 versus saline treated animals. (TIF) [file pone.0072979.s004.tif]

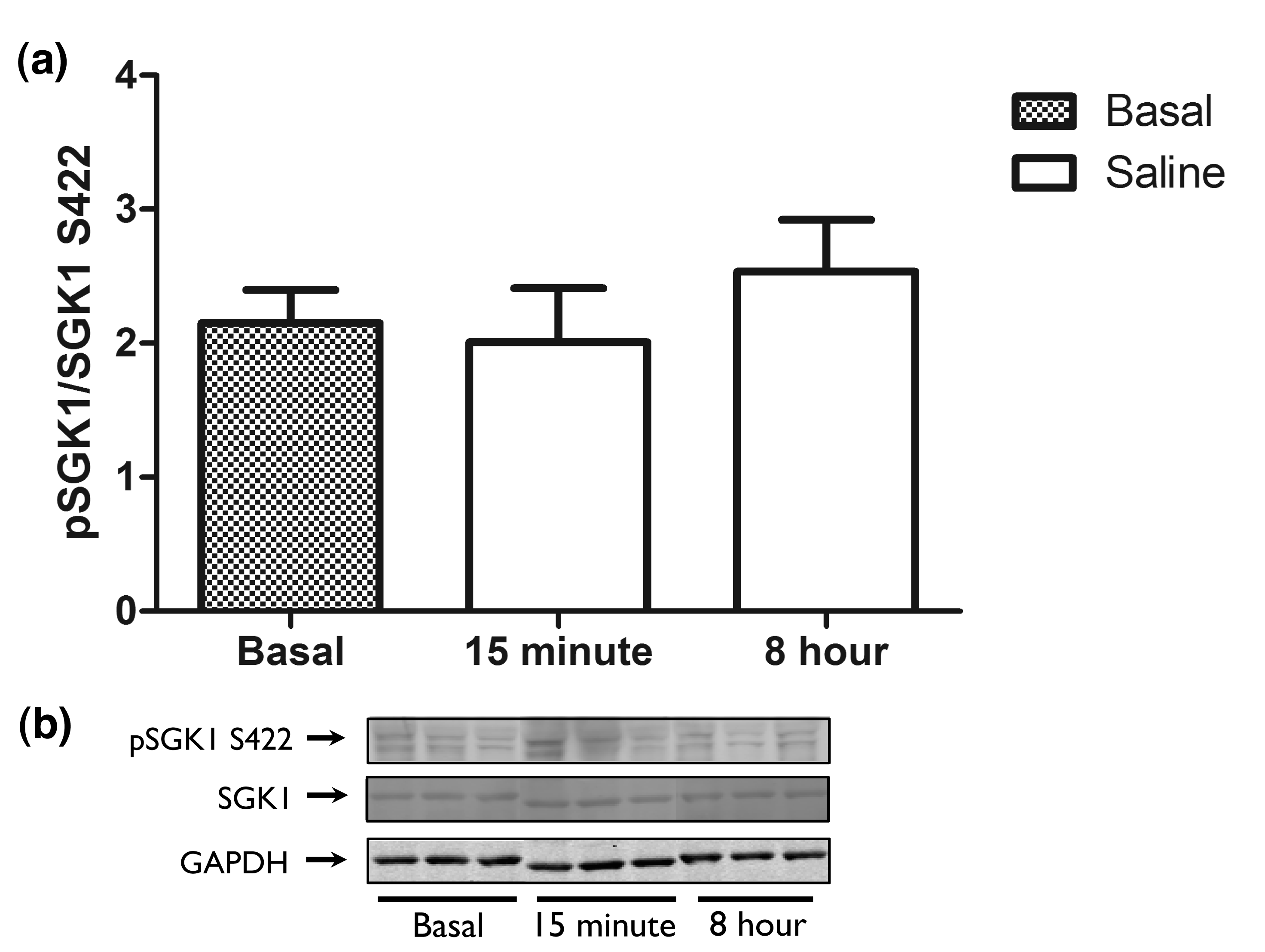

Supplement: Figure S5 — Basal versus saline treated SGK1 levels. There were no differences in SGK1 levels basally, 15 minutes following saline injection or 8 hours following saline injection. Panels show: (a) Quantification of SGK1, (b) Representative Western blot. (TIF) [file pone.0072979.s005.tif]

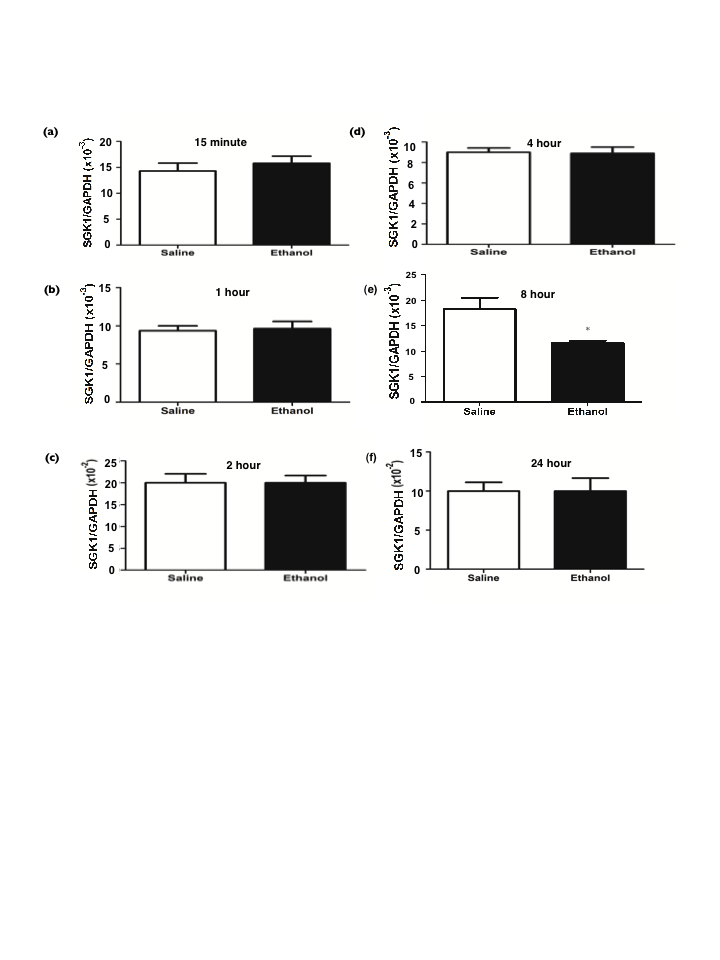

Supplement: Figure S6 — Time Course Western blot analysis of pSGK1 S422. pSGK1 S422 was significantly increased 15 minutes following ethanol versus saline administration (a). There were no significant changes in pSGK1 S422 levels at any other time point (b–f). * p < 0.05 versus saline treated animals. (TIF) [file pone.0072979.s006.tif]

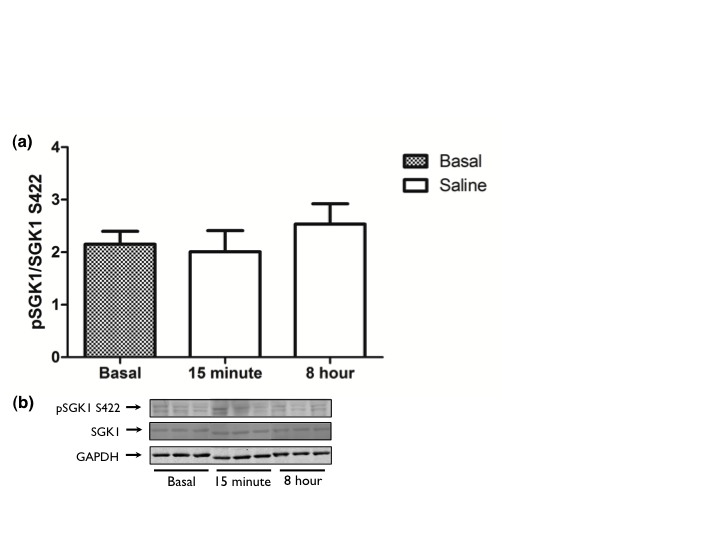

Supplement: Figure S7 — Basal versus saline treated SGK1 pS422 levels. There were no differences in SGK1 pS422 levels basally, 15 minutes following saline injection or 8 hours following saline injection. Panels show: (a) Quantification of SGK1 pS422, (b) Representative Western blot. (TIF) [file pone.0072979.s007.tif]
